# Development and Preliminary Evaluation of RR-TB GoldDx and Hr-TB GoldDx: Rapid Visual RPA-Gold Nanoparticle Assays for Detecting Rifampicin- and Isoniazid-Resistance-Associated Mutations in *Mycobacterium tuberculosis*

**DOI:** 10.3390/bios16090506

**Published:** 2026-09-10

**Authors:** Usanee Wattananandkul, Sukanya Saikaew, Sirikwan Sangboonruang, Rodjana Pongsararuk, Prapaporn Srilohasin, Bordin Butr-Indr, Sorasak Intorasoot, Chayada Sitthidet Tharinjaroen, Natthawat Semakul, Surachet Arunothong, Angkana Chaiprasert, Khajornsak Tragoolpua

**Affiliations:** 1 Division of Clinical Microbiology, Department of Medical Technology, Faculty of Associated Medical Sciences, Chiang Mai University, Chiang Mai 50200, Thailand; sukanya.saikaew@cmu.ac.th (S.S.); sirikwan.sang@cmu.ac.th (S.S.); bordin.b@cmu.ac.th (B.B.-I.); sorasak.in@cmu.ac.th (S.I.); chayada.si@cmu.ac.th (C.S.T.); khajornsak.tr@cmu.ac.th (K.T.); 2 Infectious Diseases Research Unit (IDRU), Faculty of Associated Medical Sciences, Chiang Mai University, Chiang Mai 50200, Thailand; 3 Office of Research Administration, Chiang Mai University, Chiang Mai 50200, Thailand; 4 Office of Disease Prevention and Control Region 1 (ODPC-1) Chiang Mai, Department of Disease Control, The Ministry of Public Health, Muang District, Chiang Mai 50100, Thailand; rojjj.suya@gmail.com (R.P.); lek_surachet@yahoo.com (S.A.); 5 Department of Microbiology, Faculty of Medicine Siriraj Hospital, Mahidol University, Bangkok 10700, Thailand; prapaporn.srl@mahidol.ac.th; 6 Drug Resistant Tuberculosis Research (DR-TB) Fund, Siriraj Foundation, Bangkok 10700, Thailand; angkana.cha@mahidol.ac.th; 7 Department of Chemistry, Faculty of Sciences, Chiang Mai University, Chiang Mai 50200, Thailand; natthawat.semakul@cmu.ac.th; 8 Office for Research, Faculty of Medicine Siriraj Hospital, Mahidol University, Bangkok 10700, Thailand

**Keywords:** drug-resistant tuberculosis, molecular diagnostics, point-of-care testing, recombinase polymerase amplification, gold nanoparticles, antimicrobial resistance, resource-limited settings

## Abstract

Drug-resistant tuberculosis remains a major challenge to tuberculosis control, particularly in settings with limited access to conventional molecular testing. This study developed and preliminarily evaluated RR-TB GoldDx and Hr-TB GoldDx, rapid visual assays for detecting rifampicin- and isoniazid-resistance-associated mutations in *Mycobacterium tuberculosis*. The assays combine recombinase polymerase amplification with unmodified gold nanoparticles (AuNPs) for amplification confirmation and thiol-probe-modified AuNPs for detecting resistance-associated mutations at *rpoB* codons 516, 526, and 531, *katG* codon 315, and position −15 of the *fabG1–inhA* promoter. Evaluation using DNA from 50 *M. tuberculosis* isolates yielded positive internal-control results for all samples. RR-TB GoldDx achieved accuracies of 84% and 86% against phenotypic drug-susceptibility testing and DNA sequencing, respectively, with Cohen’s kappa values of 0.68 and 0.72. Hr-TB GoldDx achieved 80% accuracy and a kappa value of 0.60 against phenotypic testing, while the *inhA* promoter and *katG* assays achieved sequencing-based accuracies of 84% and 86%, respectively. Results were available within 1 h at an estimated reagent cost of THB 500–550 (approximately USD 15–16) per test without conventional thermocycling, real-time fluorescence detection, or gel electrophoresis. These instrument-minimal assays show potential for rapid drug-resistance screening but require validation in larger, genetically diverse cohorts and direct respiratory specimens.

## 1. Introduction

Over the past decade, the global tuberculosis incidence rate declined by only 12.3% between 2015 and 2024, falling substantially short of the WHO End TB Strategy milestone of a 50% reduction by 2025, while an estimated 10.7 million people developed the disease in 2024 [1]. In the same year, approximately 390,000 people developed multidrug- or rifampicin-resistant tuberculosis (MDR/RR-TB), resulting in an estimated 150,000 deaths, while approximately 1.5 million developed isoniazid-resistant tuberculosis [1]. Rifampicin and isoniazid are key components of first-line tuberculosis treatment, and resistance to both drugs defines MDR-TB, which requires more complex and costly treatment and is associated with poorer outcomes and continued transmission [1,2,3]. Drug-resistant tuberculosis also contributes substantially to the global antimicrobial-resistance burden and can arise through the selection of resistance during inadequate or ineffective treatment or through direct transmission of resistant strains [2,3]. Despite advances in treatment, a substantial gap persists between the estimated burden of MDR/RR-TB and the number of affected individuals enrolled in appropriate treatment; in 2024, only 164,545 of the estimated 390,000 incident cases were enrolled in MDR/RR-TB treatment [1]. These diagnostic and treatment gaps underscore the need for rapid, accessible methods for detecting resistance-associated mutations.

Conventional culture-based drug-susceptibility testing (DST) remains an important reference method for characterising resistance in *Mycobacterium tuberculosis*. However, its dependence on mycobacterial growth delays the availability of results and requires specialised biosafety facilities, laboratory infrastructure, and trained personnel [4]. WHO-recommended rapid molecular diagnostics, including Xpert MTB/RIF or Xpert MTB/RIF Ultra, line-probe assays, and targeted next-generation sequencing, have substantially reduced the time required to detect tuberculosis and drug resistance [4]. In addition to these WHO-recommended diagnostics, molecular assays based on multiplex real-time PCR combined with melting analysis have been developed for the detection of tuberculosis and rifampicin- and isoniazid-resistance-associated mutations in *M. tuberculosis* [5,6,7]. These approaches have demonstrated high diagnostic sensitivity, with relatively low per-test costs of approximately USD 7–10, while markedly reducing diagnostic turnaround time to a few hours or approximately 4.5 h. However, they require fluorescent real-time PCR instrumentation and interpretation of melting-curve profiles. Despite these advances, implementation of these rapid molecular diagnostics may be limited by instrument and consumable costs, technical complexity, maintenance requirements, and dependence on stable laboratory infrastructure [4]. In addition, some commonly implemented rapid molecular assays primarily provide rifampicin-resistance results, whereas broader detection of resistance to isoniazid and other drugs may require supplementary or follow-on testing [4]. These constraints can limit timely access to comprehensive molecular DST, particularly in decentralised healthcare facilities and resource-limited settings.

Rifampicin resistance is predominantly associated with mutations within the rifampicin resistance-determining region (RRDR) of the *rpoB* gene. Codons 435, 445, and 450 according to the current *M. tuberculosis* numbering system—historically referred to as codons 516, 526, and 531, respectively—represent major mutational hotspots associated with rifampicin resistance [8,9]. Isoniazid resistance is genetically more heterogeneous but is frequently associated with mutations at *katG* codon 315 and within the *fabG1–inhA* promoter, particularly at position −15 [6,8,10]. Mutations in *katG* can impair activation of the isoniazid prodrug, which commonly leads to high-level isoniazid resistance, whereas mutations in the *fabG1–inhA* promoter can increase *inhA* expression and reduce susceptibility to isoniazid [6,10]. These major resistance-determining loci therefore represent important molecular targets for the rapid detection of rifampicin- and isoniazid-resistant *M. tuberculosis*.

Recombinase polymerase amplification (RPA) is an isothermal nucleic-acid amplification method that enables rapid amplification at a relatively low and constant temperature, typically 37–42 °C, thereby reducing dependence on conventional thermocycling instrumentation [11,12]. Its rapid reaction kinetics, simple workflow, and compatibility with multiple endpoint-detection formats make RPA attractive for decentralised molecular testing [11,12]. Other isothermal amplification approaches, such as loop-mediated isothermal amplification (LAMP), have also been applied to the detection of multidrug-resistant tuberculosis by targeting resistance-associated mutations in *rpoB*, *katG*, and *inhA* [13]. Although LAMP provides rapid amplification without conventional thermocycling, it generally requires multiple primers with relatively complex primer design and operates at a higher reaction temperature, typically around 60–65 °C.

More recently, an allele-specific RPA assay combined with SYBR Green I colourimetric detection was developed for rapid detection of multidrug-resistant tuberculosis. The assay targeted major resistance-associated mutations in *rpoB* codons 516, 526, and 531 and *katG* codon 315 and enabled naked-eye interpretation within approximately 30–40 min [14]. Although the assay showed 100% sensitivity and specificity for these targeted mutations compared with DNA sequencing, its detection range was restricted to four predefined mutation sites. Moreover, when evaluated against phenotypic DST, its ability to identify isoniazid resistance was affected by the limited mutation coverage, as isoniazid resistance may arise from mutations outside *katG* codon 315, including mutations in the *fabG1–inhA* promoter region.

Gold nanoparticles (AuNPs) provide an instrument-minimal optical readout because changes in nanoparticle dispersion and aggregation alter their localised surface plasmon resonance, producing distinct red-to-purple or blue colour changes that can be interpreted visually [15,16]. Thiol-modified oligonucleotide probes can be immobilised on AuNP surfaces through strong sulphur–gold interactions to enable sequence-specific target recognition [16,17]. In contrast, single-stranded amplification products can adsorb onto unmodified AuNPs and protect them against salt-induced aggregation [18]. Integrating RPA with AuNP-based colourimetric detection may therefore provide rapid amplification and visually interpretable screening of resistance-associated mutations without real-time fluorescence analysis or gel electrophoresis. A previous proof-of-concept study combined LAMP with thiol-modified AuNP probes for colourimetric detection of an allele-specific mutation associated with rifampicin resistance in *M. tuberculosis* [19]. Although this study demonstrated the feasibility of coupling isothermal amplification with AuNP-based visual detection for drug-resistance genotyping, it targeted a single rifampicin-resistance-associated mutation and was evaluated using a limited validation set. This assay was based on LAMP rather than RPA and did not address the complementary development of assays for both rifampicin- and isoniazid-resistance-associated mutations. Moreover, the assay did not incorporate an internal amplification control to confirm successful target amplification before interpretation of the drug-resistance result, making it difficult to distinguish a true negative result from amplification failure.

Despite advances in isothermal amplification and AuNP-based biosensing, there remains a need for an integrated visual platform that both confirms target amplification and screens for major mutations associated with rifampicin and isoniazid resistance. Therefore, this study developed and preliminarily evaluated two complementary assays, RR-TB GoldDx and Hr-TB GoldDx, for the rapid detection of resistance-associated mutations in *M. tuberculosis*. Each assay incorporates an internal-control test based on RPA and unmodified AuNPs to confirm successful amplification, together with drug-resistance tests using thiol-probe-functionalised AuNPs for wild-type sequence recognition. RR-TB GoldDx targets *rpoB* codons 435, 445, and 450, historically referred to as codons 516, 526, and 531, respectively, whereas Hr-TB GoldDx targets *katG* codon 315 and position −15 of the *fabG1–inhA* promoter. The assays were optimised and preliminarily evaluated using DNA from clinical *M. tuberculosis* isolates characterised by phenotypic DST and DNA sequencing. Agreement with phenotypic DST and DNA sequencing, turnaround time, and estimated reagent costs were assessed to determine the potential of these assays as rapid, instrument-minimal platforms for drug-resistance screening.

## 2. Materials and Methods

### 2.1. DNA Samples Used in This Study

Genomic DNA samples from clinical *Mycobacterium tuberculosis* isolates were obtained from two sources. First, 30 clinical *M. tuberculosis* isolates were obtained from the Office of Disease Prevention and Control Region 1 (ODPC-1), Chiang Mai, Thailand. The isolates had been recovered from patients’ sputum specimens and processed using standard mycobacterial culture procedures. Briefly, sputum specimens were decontaminated and liquefied using the N-acetyl-L-cysteine–sodium hydroxide (NALC–NaOH) method. The processed pellets were inoculated onto Löwenstein–Jensen (LJ) solid medium or into the BACTEC MGIT 960 liquid culture system (Becton, Dickinson and Company, Franklin Lakes, NJ, USA) and incubated at 37 °C for up to 8 weeks.

Cultures positive for mycobacterial growth were initially examined for membership in the *Mycobacterium tuberculosis* complex (MTC) using the STANDARD Q TB MPT64 Ag test (SD Biosensor, Suwon, Republic of Korea). Species-level identification and differentiation between MTC and nontuberculous mycobacteria (NTM) were subsequently performed using the REBA Myco-ID assay (YD Diagnostics, Yongin, Republic of Korea), according to the manufacturer’s instructions. Genomic DNA from confirmed *M. tuberculosis* isolates was then extracted using the extraction reagents supplied with the Anyplex™ MTB/NTM Real-Time Detection assay (Seegene Inc., Seoul, Republic of Korea), according to the manufacturer’s instructions, and stored at −20 °C until use.

In addition, genomic DNA samples from 20 clinical *M. tuberculosis* isolates were obtained from the Drug-Resistant Tuberculosis Research Fund, Siriraj Foundation, Mahidol University, Bangkok, Thailand. Genomic DNA was extracted from these isolates using the cetyltrimethylammonium bromide (CTAB) method.

A total of 50 genomic DNA samples from clinical *M. tuberculosis* isolates were included in the evaluation of the RR-TB GoldDx and Hr-TB GoldDx assays. The corresponding isolates had previously been characterised by phenotypic DST using the agar proportion method and by DNA sequencing of *rpoB*, *katG*, and the *fabG1–inhA* promoter region. Phenotypic DST and DNA sequencing were used as comparator methods for assay performance evaluation.

### 2.2. Design of RPA Primers and Wild-Type Thiol Probes

Recombinase polymerase amplification (RPA) primers were designed to amplify the *rpoB* and *katG* genes and the *fabG1–inhA* promoter region of *Mycobacterium tuberculosis* H37Rv (GenBank accession no. NC_000962.3). Primer design was performed according to the recommendations in the TwistAmp^®^ Basic Kit assay design manual (TwistDx, Cambridge, UK). Primer-BLAST (National Center for Biotechnology Information, NCBI) was used to design primers 25–27 base pairs (bp) in length and to evaluate their specificity in silico against members of the *M. tuberculosis* complex (MTBC). Primer pairs were designed to generate amplicons of approximately 200–360 bp (Table 1). All primers were synthesised by Integrated DNA Technologies, Inc. (Coralville, IA, USA).

Wild-type thiol-modified oligonucleotide probes were designed for RR-TB GoldDx and Hr-TB GoldDx to detect loss of wild-type sequence recognition at major rifampicin- and isoniazid-resistance-associated loci. The RR-TB GoldDx probes targeted *rpoB* codons 435, 445, and 450 according to the current *M. tuberculosis* numbering system, corresponding to the historically used codons 516, 526, and 531, respectively. The Hr-TB GoldDx probes targeted *katG* codon 315 and position −15 of the *fabG1–inhA* promoter. Each probe contained the corresponding wild-type sequence and was modified with a 5′-terminal thiol group to facilitate stable attachment to the gold nanoparticle (AuNP) surface through strong sulphur–gold interactions [20]. All thiol-modified probes were synthesised by GenScript Biotech Corporation (Piscataway, NJ, USA).

### 2.3. Recombinase Polymerase Amplification

RPA reactions were performed in a final volume of 25 µL using the TwistAmp^®^ Basic Kit (TwistDx, Cambridge, UK). Each reaction contained primer-free rehydration buffer, target-specific forward and reverse primers, 100 ng of synthetic DNA template, and sterile distilled water (SDW). The final concentration of each forward and reverse primer was 0.56 µM for amplification of *rpoB* and *katG* and 0.48 µM for amplification of the *fabG1–inhA* promoter. Magnesium acetate (MgOAc) was added to initiate amplification at final concentrations of 10.08 mM for *rpoB* and *katG* and 12.09 mM for the *fabG1–inhA* promoter.

After the addition of MgOAc, the reaction mixtures were thoroughly mixed and briefly centrifuged for 10 s. The *rpoB* target was amplified at 38 °C for 25 min, whereas *katG* and the *fabG1–inhA* promoter targets were amplified at 39 °C for 20 min using a SensoQuest Labcycler thermal cycler (SensoQuest GmbH, Göttingen, Germany). After 4 min of incubation, the reaction tubes were gently vortexed and centrifuged for 10 s, after which amplification was continued under the target-specific optimal temperature and time conditions.

### 2.4. Purification of Amplified RPA Products

The amplified RPA products were purified using the MAGTEC™ Neoclean™ DNA kit (Bioentist, Bangkok, Thailand) according to the manufacturer’s instructions. Briefly, 25 µL of each RPA product was mixed with 45 µL of MAGTEC™ Neoclean™ DNA reagent and incubated at room temperature for 5 min to allow DNA binding to the magnetic particles. The magnetic particles were then separated using a magnetic stand, and the supernatant was discarded. The particles were washed twice with 200 µL of 70% ethanol and air-dried at room temperature for 10 min. The purified DNA was eluted in 40 µL of sterile distilled water (SDW), followed by magnetic separation to remove the particles. The purified RPA products were transferred to new tubes and subsequently analysed by AuNP-based detection. All RPA products were also subjected to 2% (*w*/*v*) agarose gel electrophoresis at 100 V for 40 min to confirm target-specific amplification [21].

### 2.5. Preparation of Citrate-Capped AuNPs

Citrate-capped gold nanoparticles (AuNPs) were prepared using a sodium citrate reduction method, with minor modifications from a previously described protocol [21]. Briefly, 100 mL of 1 mM chloroauric acid (HAuCl_4_) solution was heated to boiling under vigorous stirring, followed by the rapid addition of 10 mL of 38.8 mM sodium citrate solution. The reaction mixture was continuously heated and stirred for 15 min until its colour changed from pale yellow to wine red, indicating the formation of citrate-capped AuNPs. The resulting colloidal AuNP suspension was cooled to room temperature and stirred for an additional 10 min before storage at 4 °C until use. The physicochemical characteristics of the synthesised AuNPs have been reported previously in the TB-GoldDx study [21].

### 2.6. Optimisation of the Internal-Control Tests Using Unmodified AuNPs

The internal-control tests were optimised to confirm successful RPA amplification of the *rpoB*, *katG*, and the *fabG1–inhA* promoter targets using citrate-capped unmodified AuNPs prepared as described previously [21]. Briefly, 12.5 µL of each purified RPA product was heat-denatured at 95 °C for 10 min to generate single-stranded DNA (ssDNA), followed by the addition of 37.5 µL of unmodified AuNP suspension. Magnesium chloride (MgCl_2_) was subsequently added at final concentrations ranging from 0 to 1.0 mM to determine the concentration that provided the clearest discrimination between reactions containing amplified target DNA and no-template controls.

The results were initially interpreted visually. Reactions containing amplified ssDNA remained red because adsorption of ssDNA onto the AuNP surfaces protected the nanoparticles against salt-induced aggregation. In contrast, reactions without amplified DNA underwent MgCl_2_-induced aggregation and changed from red to purple or blue. The colour responses were further assessed by ultraviolet–visible (UV–Vis) spectrophotometry. Dispersed AuNPs showed predominant absorbance near 530 nm, whereas aggregated AuNPs exhibited increased absorbance near 630 nm. The absorbance ratio at 530 and 630 nm (A_530_/A_630_) was used to support selection of the optimal MgCl_2_ concentration for each target.

The characteristics of AuNPs following the colourimetric reaction, including particle size and polydispersity index (PDI) corresponding to the colour change, were determined via dynamic light scattering (DLS) using a Zetasizer (Malvern Instruments, Worcestershire, UK). In addition, scanning transmission electron microscopy (STEM) was performed to investigate the dispersion, aggregation, and morphology of the AuNPs after the colourimetric reaction. Briefly, a drop of each sample was deposited onto a carbon-coated copper grid and air-dried prior to imaging using a JSM-IT800 Ultrahigh Resolution Field Emission SEM (JEOL, Peabody, MA, USA) [21].

### 2.7. Preparation of Thiol-Probe-Modified AuNPs

Thiol-probe-modified AuNPs were prepared by functionalising citrate-capped AuNPs with thiol-labelled wild-type oligonucleotide probes targeting *rpoB*, *katG*, and the *fabG1–inhA* promoter. For RR-TB GoldDx, Thiol-*rpoB* 516 wt, Thiol-*rpoB* 526 wt, and Thiol-*rpoB* 531 wt probes were used at final concentrations of 16, 16, and 10 µM, respectively. For Hr-TB GoldDx, Thiol-*katG* 315 wt and Thiol-*inhA* −15 wt probes were each used at a final concentration of 10 µM.

Each thiol-modified probe was mixed with tris(2-carboxyethyl) phosphine (TCEP) to obtain final TCEP and probe concentrations of 0.1 mM and the target-specific concentration indicated above, respectively. The volume was adjusted with sterile distilled water (SDW) and the mixture was incubated at room temperature for 10 min to reduce disulphide bonds and generate free thiol groups. Citrate-capped AuNPs were then added at probe/TCEP mixture-to-AuNP volume ratios of 1:3.25 for the RR-TB GoldDx probes and 1:4 for the Hr-TB GoldDx probes. The mixtures were incubated at room temperature for a further 10 min to allow attachment of the thiol-modified probes to the AuNP surfaces. The resulting probe-functionalised AuNPs were subsequently used in the drug-resistance detection assays.

### 2.8. Optimisation of the Drug-Resistance Tests Using Thiol-Probe-Modified AuNPs

The RR-TB GoldDx and Hr-TB GoldDx drug-resistance tests were optimised for detecting loss of wild-type sequence recognition at the selected resistance-associated loci using the prepared thiol-probe-modified AuNPs. Briefly, 12.5 µL of each purified RPA product was heat-denatured at 95 °C for 10 min to generate single-stranded DNA (ssDNA), followed by the addition of 37.5 µL of the corresponding probe-functionalised AuNP suspension. Magnesium chloride (MgCl_2_) was subsequently added at final concentrations ranging from 0 to 1.8 mM to determine the concentration that provided the clearest discrimination between wild-type and mutant targets.

The assay results were initially interpreted visually. Reactions containing wild-type target sequences remained red because complementary hybridisation between the ssDNA and the AuNP-bound wild-type probes stabilised the nanoparticles against salt-induced aggregation. In contrast, mutant targets with mismatches within the probe-binding regions showed reduced hybridisation, resulting in MgCl_2_-induced aggregation and a colour change from red to purple or blue. The colour responses were further evaluated by ultraviolet–visible (UV–Vis) spectrophotometry. Dispersed AuNPs showed predominant absorbance near 530 nm, whereas aggregated AuNPs exhibited increased absorbance near 630 nm. The absorbance ratio at 530 and 630 nm (A_530_/A_630_) was used to support selection of the optimal MgCl_2_ concentration for each probe-target system. In addition, the same methods described in Section 2.6 were applied to determine the particle size and PDI of the AuNPs and to visualise their dispersion and aggregation corresponding to the observed colour changes.

### 2.9. Evaluation of the RR-TB GoldDx and Hr-TB GoldDx Assays

The diagnostic performance of the RR-TB GoldDx and Hr-TB GoldDx assays was evaluated in a blinded manner using genomic DNA from 50 clinical *M. tuberculosis* isolates characterised by phenotypic DST and DNA sequencing. RR-TB GoldDx results were compared with phenotypic rifampicin DST and *rpoB* sequencing, whereas Hr-TB GoldDx results were compared with phenotypic isoniazid DST and sequencing of *katG* and the *fabG1–inhA* promoter.

Sensitivity, specificity, positive predictive value (PPV), negative predictive value (NPV), and overall accuracy were calculated with 95% confidence intervals (CIs) using the MedCalc Diagnostic Test Evaluation calculator, version 23.7.1 [22]. Agreement between each GoldDx assay and its corresponding comparator method was assessed using Cohen’s kappa coefficient in IBM SPSS Statistics, version 30.0 (IBM Corp., Armonk, NY, USA). Kappa values were interpreted as follows: ≤0, no agreement; 0.01–0.20, slight agreement; 0.21–0.40, fair agreement; 0.41–0.60, moderate agreement; 0.61–0.80, substantial agreement; and 0.81–1.00, almost perfect agreement [23].

## 3. Results

### 3.1. Design and Detection Principles of the RR-TB GoldDx and Hr-TB GoldDx Assays

**Figure 1:**
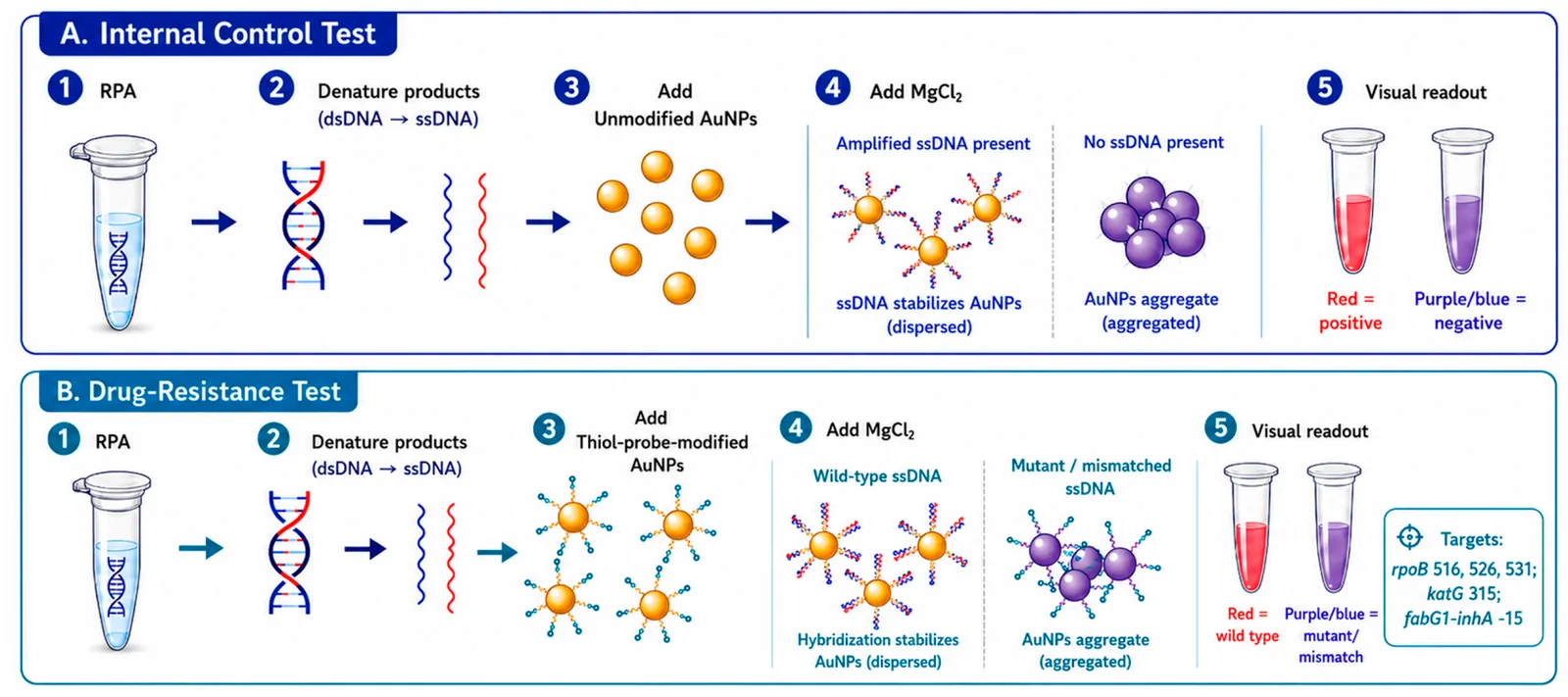
**Detection principles of the RR-TB GoldDx and Hr-TB GoldDx assays.** The platform comprises two complementary tests. (**A**) **Internal-control test:** Following RPA and thermal denaturation, single-stranded amplicons adsorb onto unmodified AuNPs and protect them against MgCl_2_-induced aggregation, maintaining a red colour. In the absence of amplified products, the AuNPs aggregate and change to purple or blue. (**B**) **Drug-resistance test:** Thiol-probe-functionalised AuNPs recognise wild-type sequences within the targeted resistance-associated regions. Complementary hybridisation stabilises the AuNPs and maintains the red colour, whereas mutations or mismatches within the probe-binding region reduce hybridisation and permit MgCl_2_-induced aggregation, producing a purple or blue colour. Results are interpreted visually without real-time fluorescence detection or gel electrophoresis. The graphical elements were generated with assistance from ChatGPT image-generation tools (OpenAI) and were subsequently reviewed, edited, and verified by the authors. This figure is a conceptual illustration and does not represent experimental data.

RR-TB GoldDx and Hr-TB GoldDx were designed as visually interpreted assays combining RPA with the salt-induced aggregation properties of AuNPs. Each assay consisted of two complementary components: (i) an internal-control test, which confirmed successful amplification of the target region, and (ii) a drug-resistance test, which assessed the presence or absence of the corresponding wild-type sequence. Following RPA amplification at 38 °C for 25 min for *rpoB* and at 39 °C for 20 min for *katG* and the *fabG1–inhA* promoter, the resulting double-stranded DNA products were thermally denatured to generate ssDNA before incubation with either unmodified or thiol-probe-functionalised AuNPs. The colour response was then interpreted visually following the addition of MgCl_2_ (Figure 1).

In the internal-control test, unmodified AuNPs were used to confirm the presence of RPA products. In samples containing the target amplicon, the denatured ssDNA adsorbed onto the AuNP surface and increased nanoparticle stability against MgCl_2_-induced aggregation. The AuNPs therefore remained dispersed and retained their red colour, indicating successful amplification. In contrast, in the absence of an RPA product, no *ss*DNA was available to stabilise the nanoparticles. Addition of MgCl_2_ consequently induced AuNP aggregation, producing a purple-to-blue colour and indicating an invalid or negative amplification reaction. This internal-control step was incorporated to distinguish a true loss of wild-type recognition in the drug-resistance test from an apparent negative result caused by amplification failure (Figure 1).

**Figure 2:**
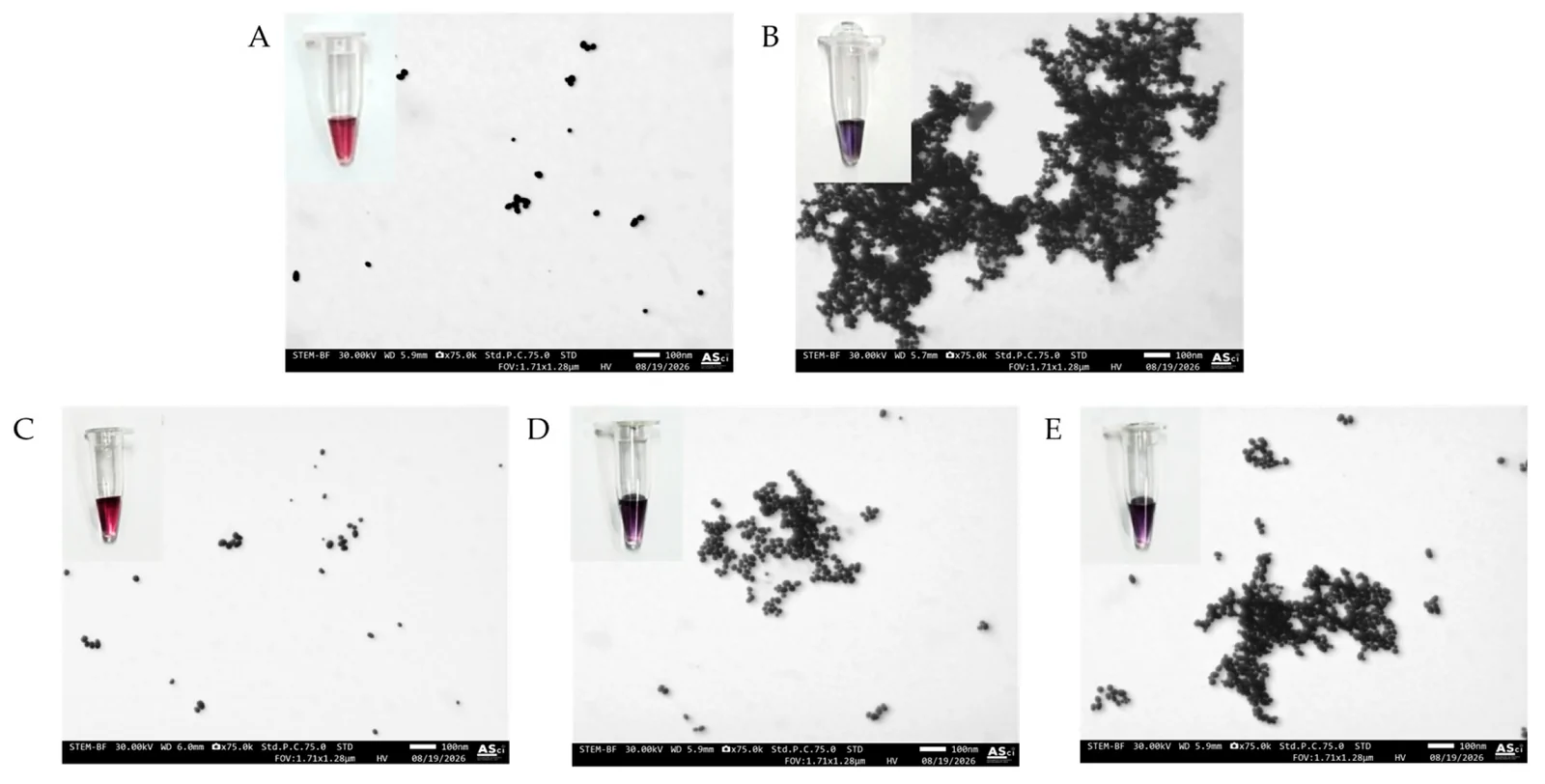
**Characteristics of AuNPs corresponding to the observed colour changes examined by scanning transmission electron microscopy (STEM).** Dispersion and aggregation behaviours of unmodified AuNPs and modified-AuNPs are shown in the internal-control test of samples (**A**) with amplified products and (**B**) without amplified products, and for the drug-resistance tests of samples containing (**C**) wild-type *fabG1–inhA* promoter sequences, (**D**) mutant *fabG1–inhA* promoter sequences, and (**E**) no target products.

The physicochemical properties and aggregation behaviour of the unmodified AuNPs corresponding to these colour changes were further evaluated by DLS and STEM. In the presence of RPA products, the reaction mixture remained red following MgCl_2_ addition, consistent with maintained AuNP dispersion. STEM imaging showed predominantly dispersed, spherical nanoparticles (Figure 2A), while DLS analysis demonstrated a mean particle diameter of approximately 44.86 ± 8.74 nm with a PDI of 0.49 ± 0.02. Conversely, in the absence of target amplification, MgCl_2_-induced aggregation resulted in a colour change to purple. STEM imaging confirmed the formation of AuNP clusters (Figure 2B), while DLS analysis showed a marked increase in the particle diameter to 1433.90 ± 795.35 nm. with a high PDI of 0.83 ± 0.15, consistent with a loss of colloidal stability and extensive nanoparticle aggregation.

In the drug-resistance test, AuNPs were functionalised with thiol-labelled oligonucleotide probes complementary to the wild-type sequences at the selected resistance-associated loci. RR-TB GoldDx contained wild-type probes targeting *rpoB* codons 516, 526, and 531, whereas Hr-TB GoldDx contained probes targeting *katG* codon 315 and position −15 of the *fabG1–inhA* promoter. When the amplified target contained the corresponding wild-type sequence, complementary hybridisation occurred between the single-stranded RPA product and the AuNP-bound probe. This hybridisation stabilised the nanoparticles against salt-induced aggregation, and the reaction remained red. By contrast, resistance-associated mutations within the probe-binding region reduced or prevented effective hybridisation. Under these conditions, the modified AuNPs aggregated after MgCl_2_ addition, resulting in a purple-to-blue colour. Thus, a red reaction indicated recognition of the wild-type sequence, whereas a purple-to-blue reaction indicated loss of wild-type recognition and the possible presence of a resistance-associated mutation.

The physicochemical properties of the probe-functionalised AuNPs corresponding to these colour responses were further evaluated by DLS and STEM. Following successful target hybridisation, the AuNPs remained in a red colloidal state, and STEM imaging showed predominantly well-dispersed, spherical nanoparticles (Figure 2C). DLS analysis demonstrated a mean particle diameter of 38.82 ± 9.67 nm and a PDI of 0.30 ± 0.02. Conversely, in the absence of effective hybridisation, MgCl_2_-induced aggregation resulted in a colour change to purple. STEM imaging confirmed AuNP clustering (Figure 2D,E), while DLS analysis demonstrated an increased mean particle diameter of 71.02 ± 12.75 nm with a PDI of 0.64 ± 0.07, consistent with reduced colloidal stability and nanoparticle aggregation.

### 3.2. Design and Selection of RPA Primers and Wild-Type Probes

Multiple candidate primer pairs were initially designed for amplification of the *rpoB*, *katG*, and *fabG1–inhA* promoter regions. Candidates were assessed for predicted amplification efficiency, minimal self-complementarity and primer–dimer formation, compatibility with RPA conditions, and inclusion of the selected resistance-associated loci. Following experimental screening, one primer pair was selected for each target, generating amplicons of 359 bp for *rpoB*, 206 bp for *katG*, and 216 bp for the *fabG1–inhA* promoter.

Primer and probe sequences were aligned with the *Mycobacterium tuberculosis* H37Rv reference genome (GenBank accession no. NC_000962.3) to confirm their target locations, sequence complementarity, and positions relative to the selected resistance-associated loci [19]. The selected primer-binding regions flanked these loci and generated amplicons containing the intended target sites. The *rpoB* amplicon encompassed codons 435, 445, and 450. The *katG* amplicon contained codon 315, whereas the *fabG1–inhA* promoter amplicon included position −15 [6,8].

Five wild-type oligonucleotide probes were designed within the amplicons: Thiol-*rpoB* 516 wt, Thiol-*rpoB* 526 wt, Thiol-*rpoB* 531 wt, Thiol-*katG* 315 wt, and Thiol-*inhA* −15 wt. Their respective lengths were 31, 33, 31, 36, and 30 nucleotides. Each probe encompassed the corresponding resistance-associated codon or promoter nucleotide to enhance discrimination between fully complementary wild-type targets and mismatched mutant targets. A 5′ thiol group was incorporated to facilitate stable attachment of the oligonucleotide probes to the AuNP surface through strong sulphur–gold interactions [17].

Because the assay relies on wild-type sequence recognition, mutations within the probe-binding regions are expected to reduce probe–target hybridisation and permit MgCl_2_-induced AuNP aggregation. Based on resistance-associated variants reported in the WHO mutation catalogue, the three *rpoB* probes theoretically cover up to 31 amino-acid substitutions across codons 435, 445, and 450 (Table A1). The Hr-TB GoldDx probes target major isoniazid resistance-associated mutations at *katG* codon 315 and position −15 of the *fabG1–inhA* promoter [6]. However, this theoretical coverage requires confirmation using well-characterised mutant isolates or synthetic targets.

### 3.3. Optimisation of the Internal-Control Tests

The internal-control tests were optimised to confirm successful RPA amplification of the *rpoB*, *katG*, and *fabG1–inhA* promoter targets using unmodified AuNPs. Following amplification, the RPA products were thermally denatured and mixed with unmodified AuNPs before the addition of MgCl_2_. MgCl_2_ concentrations ranging from 0 to 1.0 mM were evaluated to identify conditions that clearly distinguished samples containing RPA products from the no-template controls.

**Figure A1:**
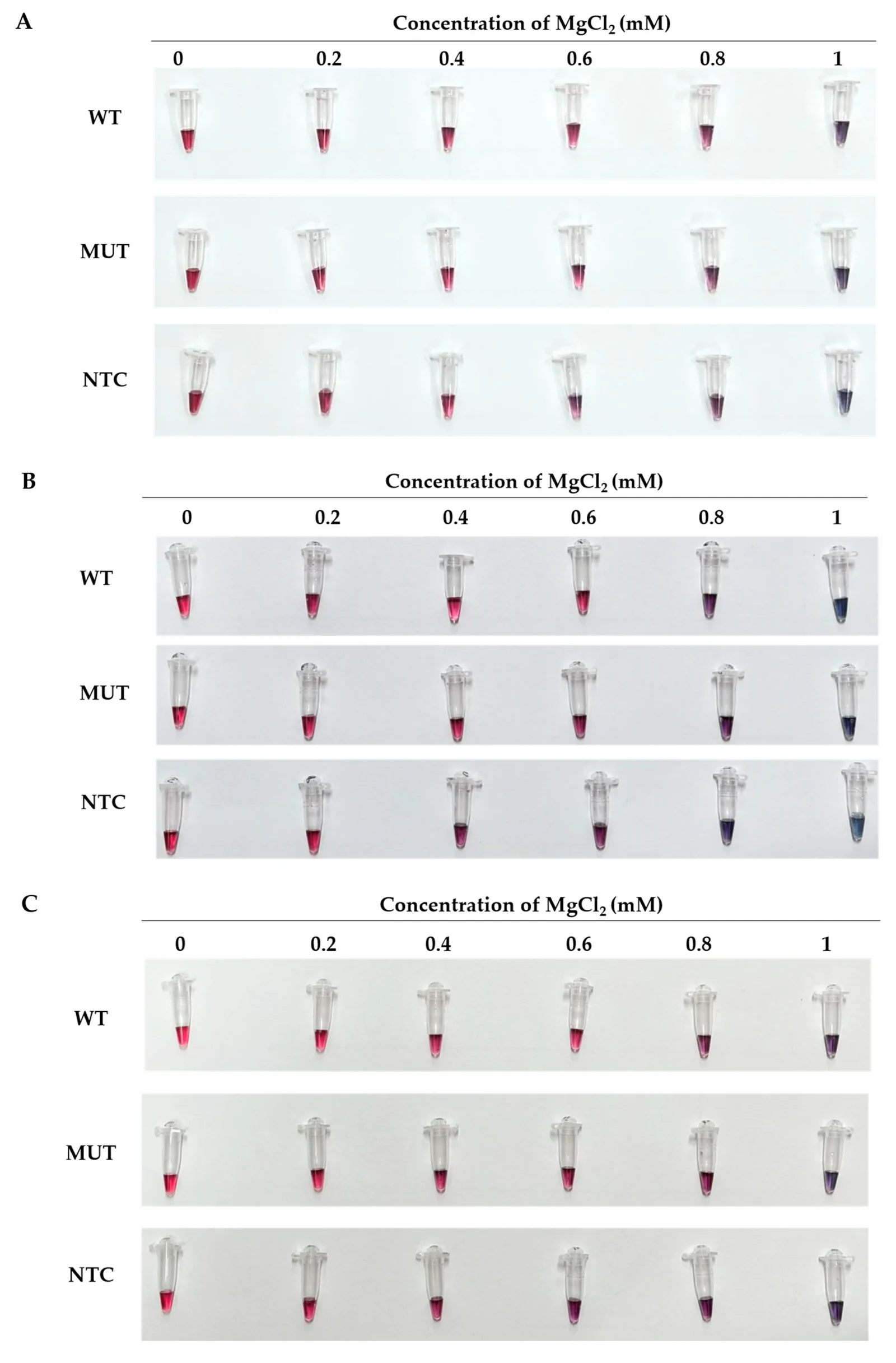
**Optimisation of MgCl_2_ concentrations for the unmodified-AuNP internal-control tests.** Visual colour responses following RPA amplification of (**A**) *rpoB*, (**B**) *katG*, and (**C**) the *fabG1–inhA* promoter at different MgCl_2_ concentrations using wild-type (WT), mutant (MUT), and a no-template control (NTC) samples. Amplified samples remained red, whereas no-template controls underwent AuNP aggregation and changed to purple.

**Figure A2:**
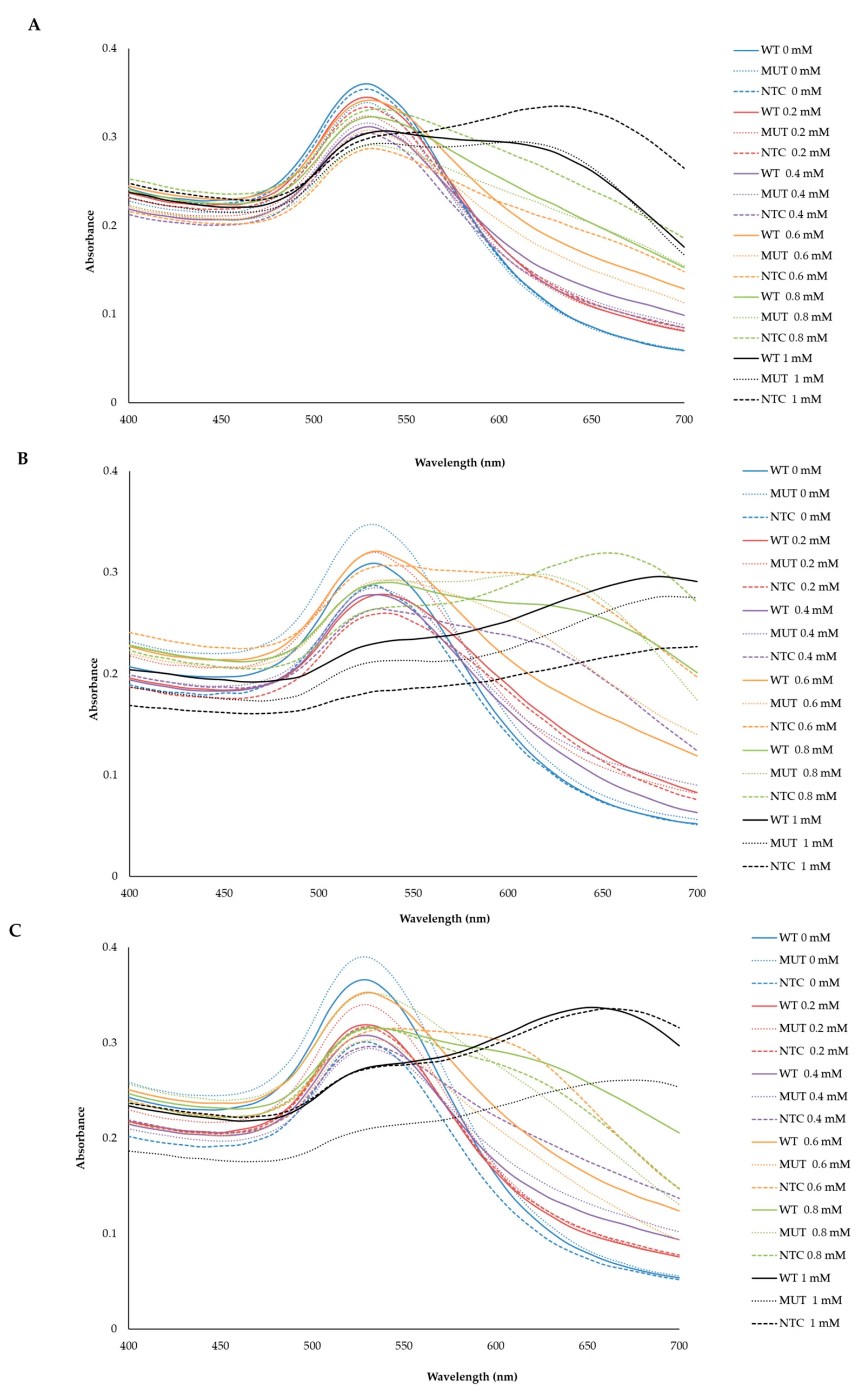
**UV–Vis characterisation of the optimised unmodified-AuNP internal-control tests.** Absorbance spectra recorded from 400 to 700 nm following RPA amplification of (**A**) *rpoB*, (**B**) *katG*, and (**C**) the *fabG1–inhA* promoter at different MgCl_2_ concentrations. The spectra are represented by different line patterns: solid lines for wild-type (WT), dotted lines for mutant (MUT), and dashed lines for the no-template control (NTC). Dispersed AuNPs exhibited predominant absorbance near 530 nm, whereas aggregation increased absorbance near 630 nm.

The clearest visual discrimination was obtained using 0.6 mM MgCl_2_ for *rpoB* and the *fabG1–inhA* promoter and 0.4 mM MgCl_2_ for *katG* (Figure A1). Under these conditions, both wild-type and mutant *M. tuberculosis* samples remained red, indicating successful target amplification, whereas the no-template controls changed to purple because of MgCl_2_-induced AuNP aggregation. UV–Vis analysis supported the visual observations: dispersed AuNPs showed predominant absorbance near 530 nm, while aggregated AuNPs exhibited increased absorbance near 630 nm (Figure A2). These conditions were therefore selected for the internal-control components of RR-TB GoldDx and Hr-TB GoldDx.

### 3.4. Optimisation of the RR-TB GoldDx Drug-Resistance Tests

The RR-TB GoldDx drug-resistance tests were optimised using wild-type thiol probes targeting *rpoB* codons 516, 526, and 531. Probe concentrations of 8–16 µM and MgCl_2_ concentrations of up to 2.0 mM were evaluated to identify conditions that provided the clearest discrimination between wild-type and mutant targets.

**Figure 3:**
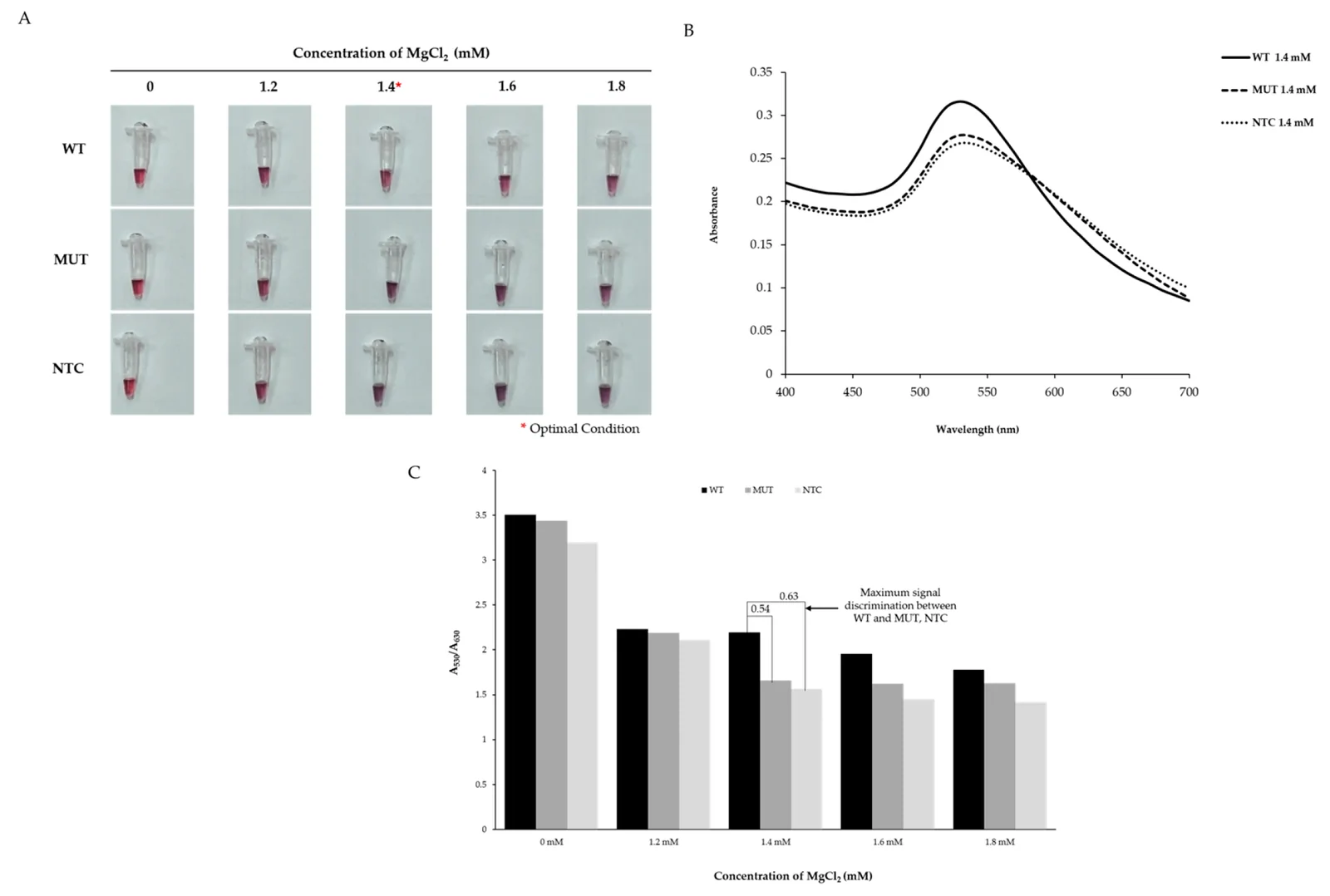
**Optimisation of the RR-TB GoldDx drug-resistance test targeting *rpoB* codon 516.** Thiol-*rpoB* 516 wt probe-functionalised AuNPs were evaluated at a final probe concentration of 16 µM using MgCl_2_ concentrations of 0, 1.2, 1.4, and 1.8 mM. (**A**) Visual colour responses of wild-type (WT), mutant (MUT), and no-template control (NTC) reactions following MgCl_2_ addition. (**B**) UV–Vis absorbance spectra recorded from 400 to 700 nm. (**C**) A_530_/A_630_ absorbance ratios at each MgCl_2_ concentration. A final MgCl_2_ concentration of 1.4 mM provided the clearest discrimination between wild-type and mutant targets.

**Figure 4:**
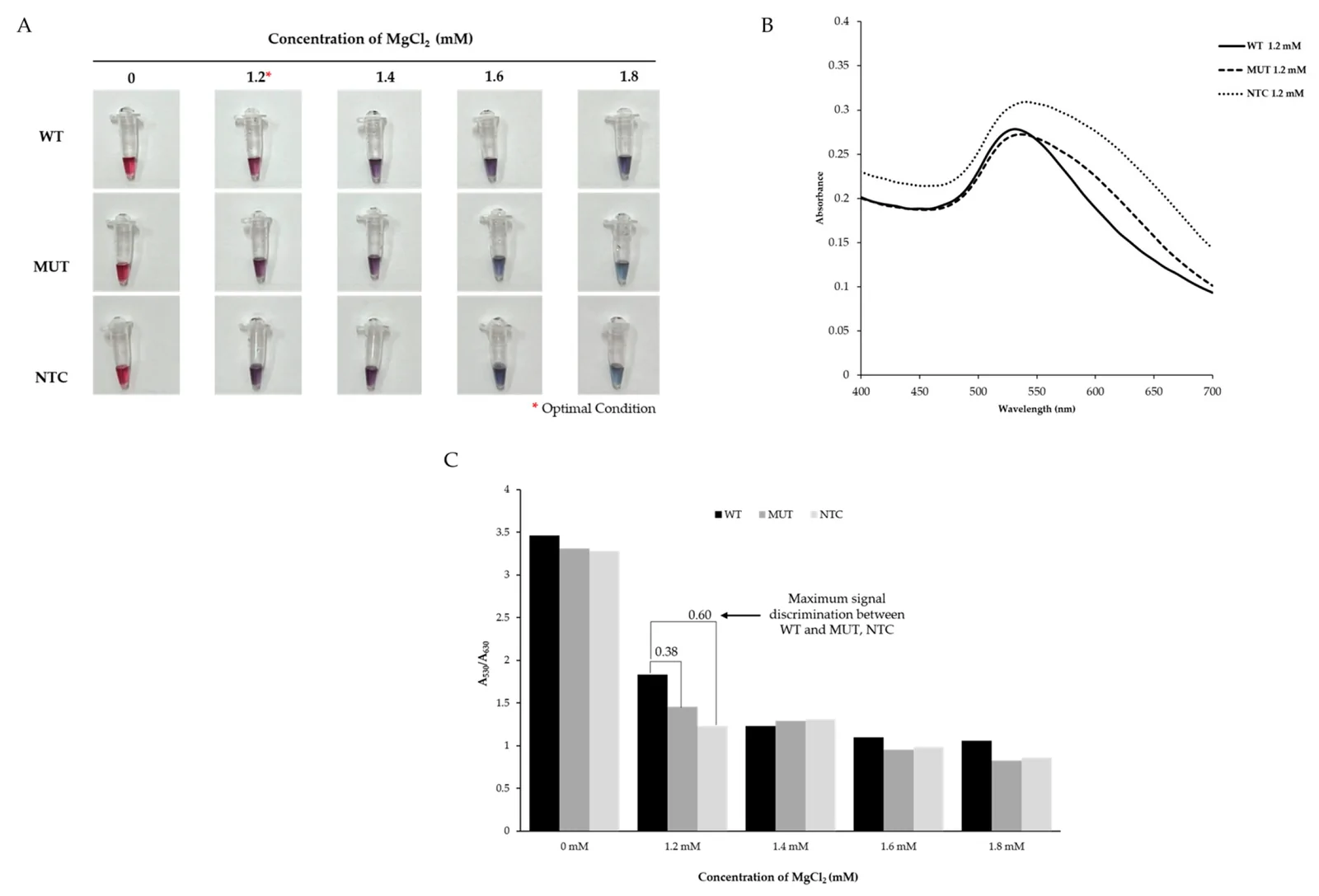
**Optimisation of the RR-TB GoldDx drug-resistance test targeting *rpoB* codon 526.** Thiol-*rpoB* 526 wt probe-functionalised AuNPs were evaluated at a final probe concentration of 16 µM using MgCl_2_ concentrations of 0, 1.2, 1.4, and 1.8 mM. (**A**) Visual colour responses of wild-type (WT), mutant (MUT), and no-template control (NTC) reactions following MgCl_2_ addition. (**B**) UV–Vis absorbance spectra recorded from 400 to 700 nm. (**C**) A_530_/A_630_ absorbance ratios at each MgCl_2_ concentration. A final MgCl_2_ concentration of 1.2 mM provided the clearest discrimination between wild-type and mutant targets.

**Figure 5:**
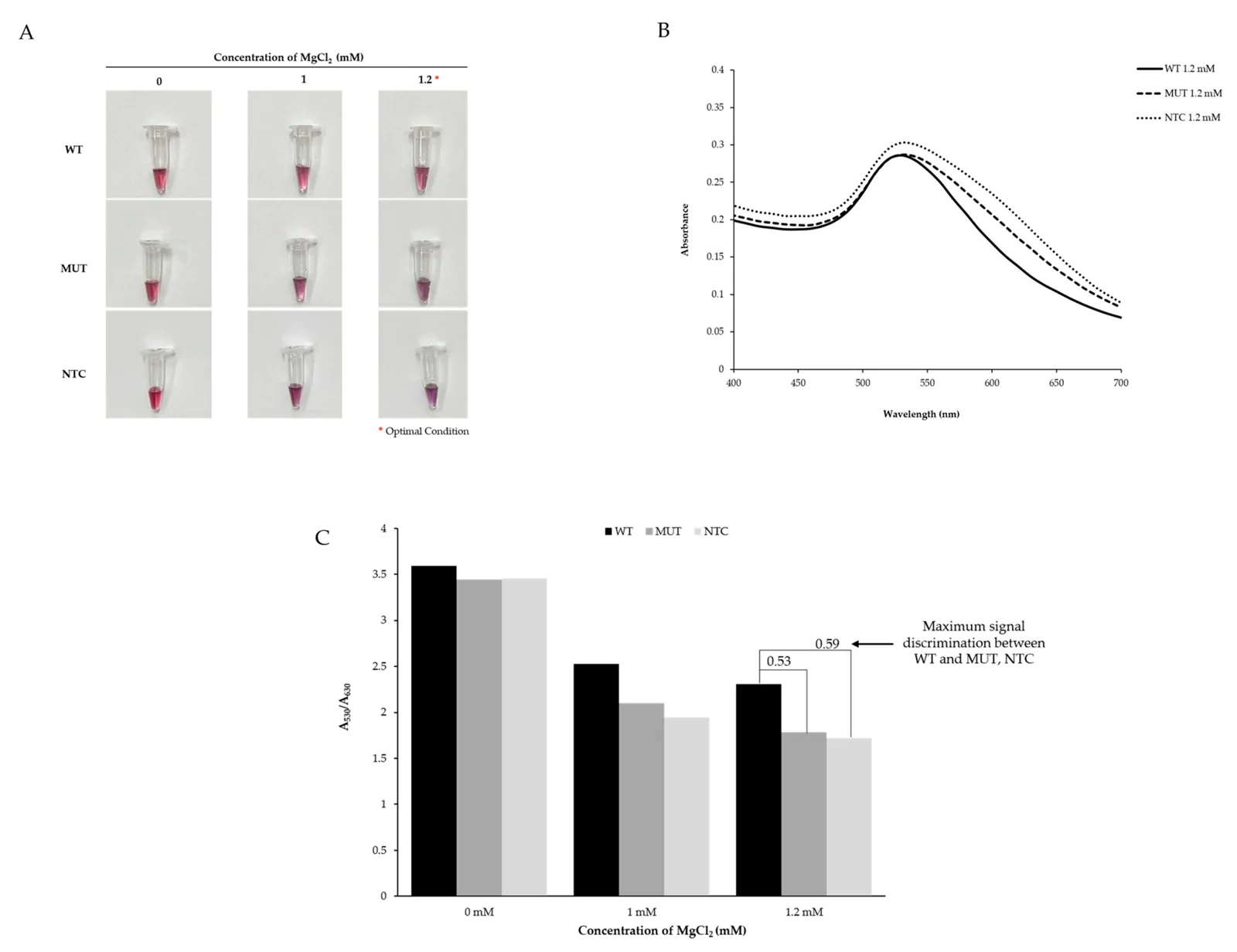
**Optimisation of the RR-TB GoldDx drug-resistance test targeting *rpoB* codon 531.** Thiol-*rpoB* 531 wt probe-functionalised AuNPs were evaluated at a final probe concentration of 8 µM using MgCl_2_ concentrations of 0, 1.2, 1.4, and 1.8 mM. (**A**) Visual colour responses of wild-type (WT), mutant (MUT), and no-template control (NCT) reactions following MgCl_2_ addition. (**B**) UV–Vis absorbance spectra recorded from 400 to 700 nm. (**C**) A_530_/A_630_ absorbance ratios at each MgCl_2_ concentration. A final MgCl_2_ concentration of 1.2 mM provided the clearest discrimination between wild-type and mutant targets.

For *rpoB* codon 516, the optimal condition was 16 µM Thiol-*rpoB* 516 wt with 1.4 mM MgCl_2_, which clearly distinguished the red wild-type reaction from the purple mutant and no-template control reactions (Figure 3). For codon 526, 16 µM Thiol-*rpoB* 526 wt with 1.2 mM MgCl_2_ provided the clearest discrimination between wild-type and mutant targets (Figure 4). For codon 531, the optimal condition was 8 µM Thiol-*rpoB* 531 wt with 1.2 mM MgCl_2_ (Figure 5).

Under the selected conditions, wild-type RPA products hybridised with the corresponding AuNP-bound probes and maintained the dispersed red colour. In contrast, mutant targets and no-template controls underwent MgCl_2_-induced aggregation and changed to purple or blue, indicating loss of wild-type probe recognition. The visual findings were supported by the corresponding UV–Vis absorbance spectra and A_530_/A_630_ ratios shown in Figure 3, Figure 4 and Figure 5.

### 3.5. Optimisation of the Hr-TB GoldDx Drug-Resistance Tests

The Hr-TB GoldDx drug-resistance tests were optimised using wild-type thiol probes targeting *katG* codon 315 and position −15 of the *fabG1–inhA* promoter. Probe concentrations of 8–16 µM and MgCl_2_ concentrations of up to 2.0 mM were evaluated to identify conditions that most clearly discriminated wild-type from mutant targets.

**Figure 6:**
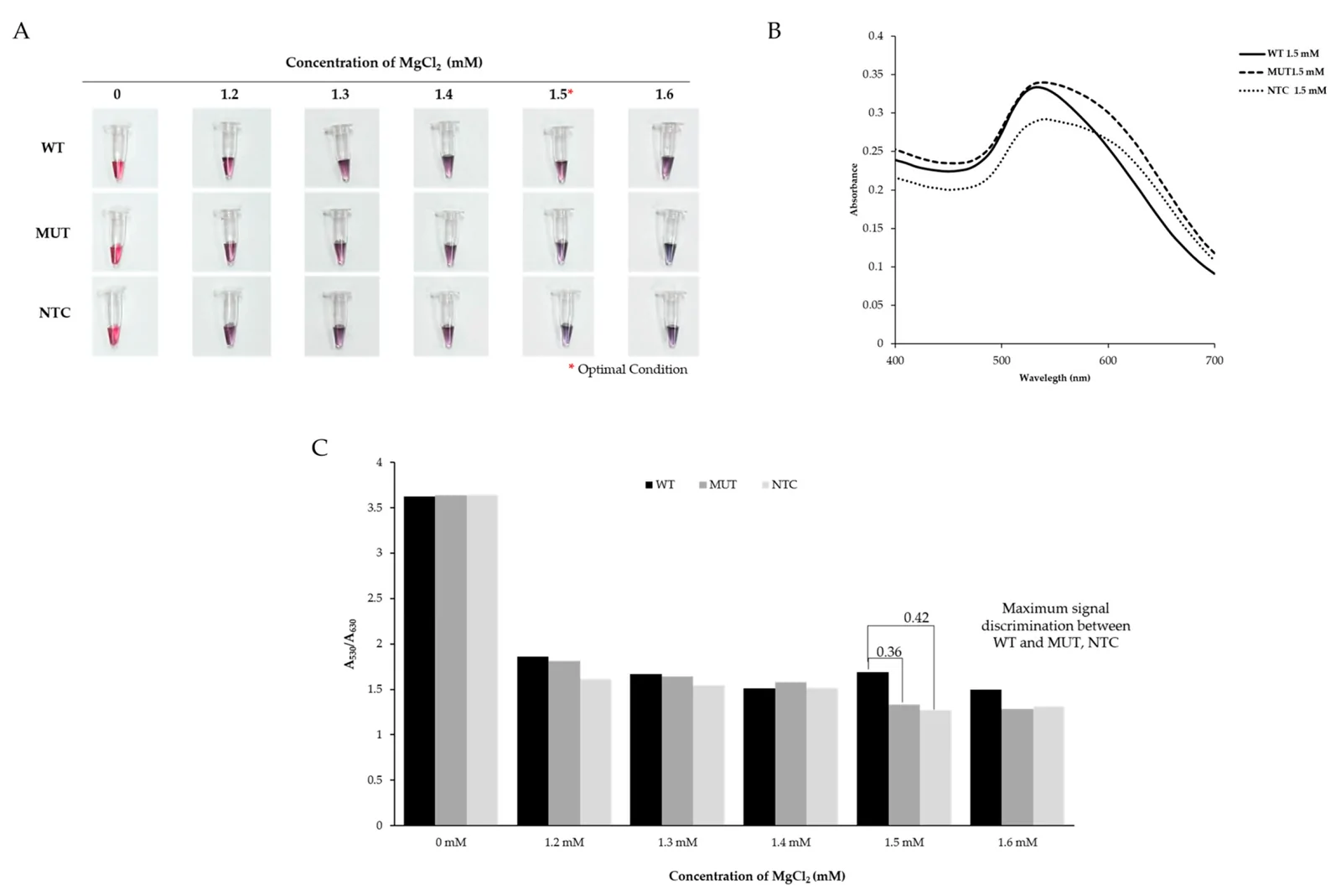
**Optimisation of the Hr-TB GoldDx drug-resistance test targeting *katG* codon 315.** Thiol-*katG* 315 wt probe-functionalised AuNPs were evaluated at a final probe concentration of 10 µM using MgCl_2_ concentrations of 0, 1.2, 1.3, 1.4, 1.5, and 1.6 mM. (**A**) Visual colour responses of wild-type (WT), mutant (MUT), and no-template control (NTC) reactions following MgCl_2_ addition. (**B**) UV–Vis absorbance spectra recorded from 400 to 700 nm. (**C**) A_530_/A_630_ absorbance ratios at each MgCl_2_ concentration. A final MgCl_2_ concentration of 1.5 mM provided the clearest discrimination between the red wild-type reaction and the aggregated purple mutant and no-template control reactions.

**Figure 7:**
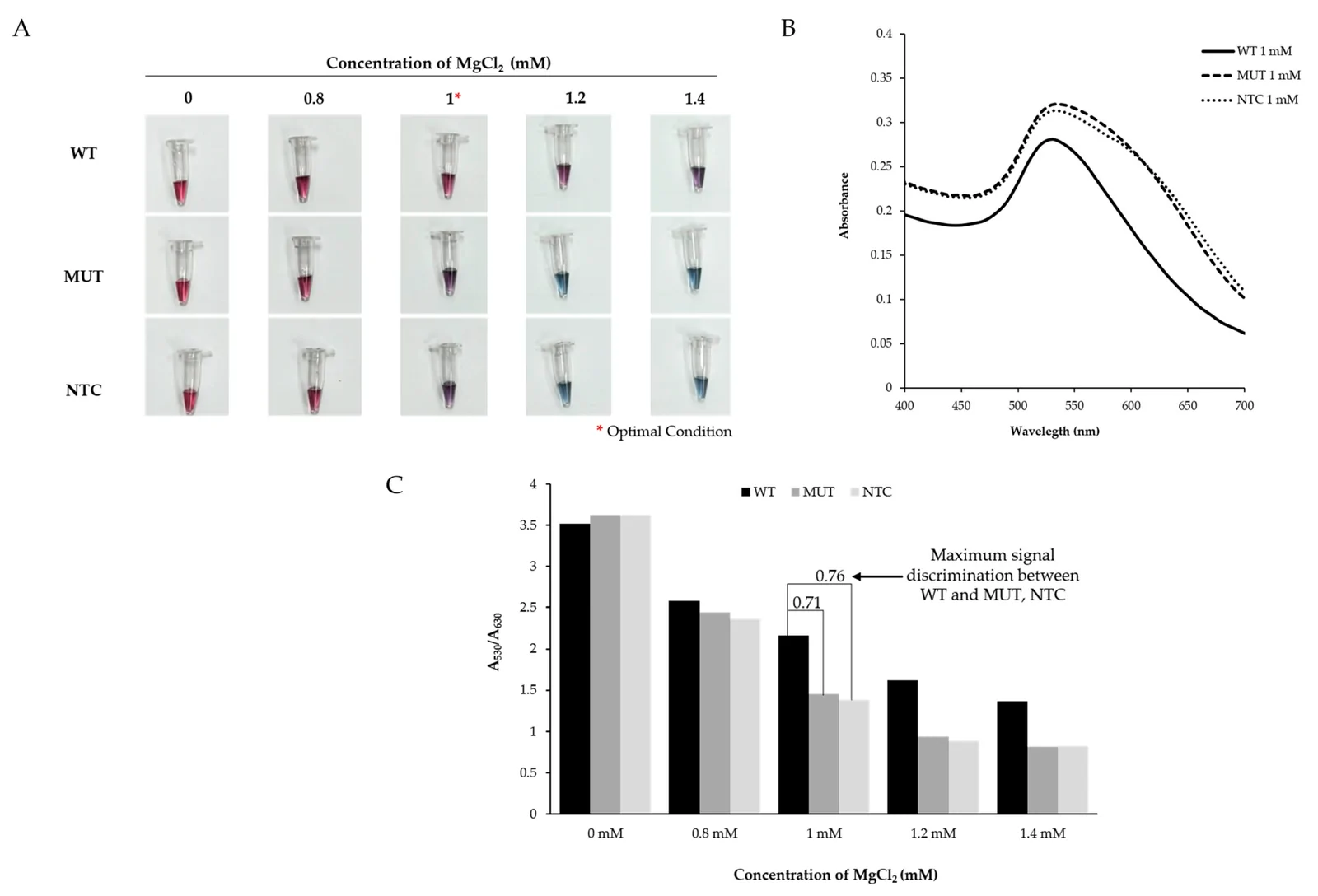
**Optimisation of the Hr-TB GoldDx drug-resistance test targeting position −15 of the *fabG1–inhA* promoter.** Thiol-*inhA* −15 wt probe-functionalised AuNPs were evaluated at a final probe concentration of 10 µM using MgCl_2_ concentrations of 0, 0.8, 1.0, 1.2, and 1.4 mM. (**A**) Visual colour responses of wild-type (WT), mutant (MUT), and no-template control (NTC) reactions following MgCl_2_ addition. (**B**) UV–Vis absorbance spectra recorded from 400 to 700 nm. (**C**) A_530_/A_630_ absorbance ratios at each MgCl_2_ concentration. A final MgCl_2_ concentration of 1.0 mM provided the clearest discrimination between wild-type and mutant targets.

For *katG* codon 315, the optimal condition was 10 µM Thiol-*katG* 315 wt with 1.5 mM MgCl_2_. Under this condition, the wild-type reaction remained red, whereas the mutant and no-template control reactions changed to purple following MgCl_2_-induced AuNP aggregation (Figure 6). For position −15 of the *fabG1–inhA* promoter, the optimal condition was 10 µM Thiol-*inhA* −15 wt with 1.0 mM MgCl_2_, which provided clear visual discrimination between wild-type and mutant targets (Figure 7).

Under the selected conditions, wild-type RPA products hybridised with the corresponding AuNP-bound probes and stabilised the nanoparticles against salt-induced aggregation. In contrast, mutations within the probe-binding regions reduced wild-type probe recognition, resulting in AuNP aggregation and a purple colour. The visual findings were supported by the corresponding UV–Vis absorbance spectra and A_530_/A_630_ ratios shown in Figure 6 and Figure 7.

### 3.6. Evaluation of RR-TB GoldDx and Hr-TB GoldDx Using Characterised M. tuberculosis Isolates

The performance of RR-TB GoldDx and Hr-TB GoldDx was evaluated in a blinded analysis using DNA from 50 clinical *M. tuberculosis* isolates. Phenotypic DST and DNA sequencing of the corresponding resistance-associated loci were used as comparator methods.

#### 3.6.1. Performance of the Internal-Control Tests

**Figure 8:**
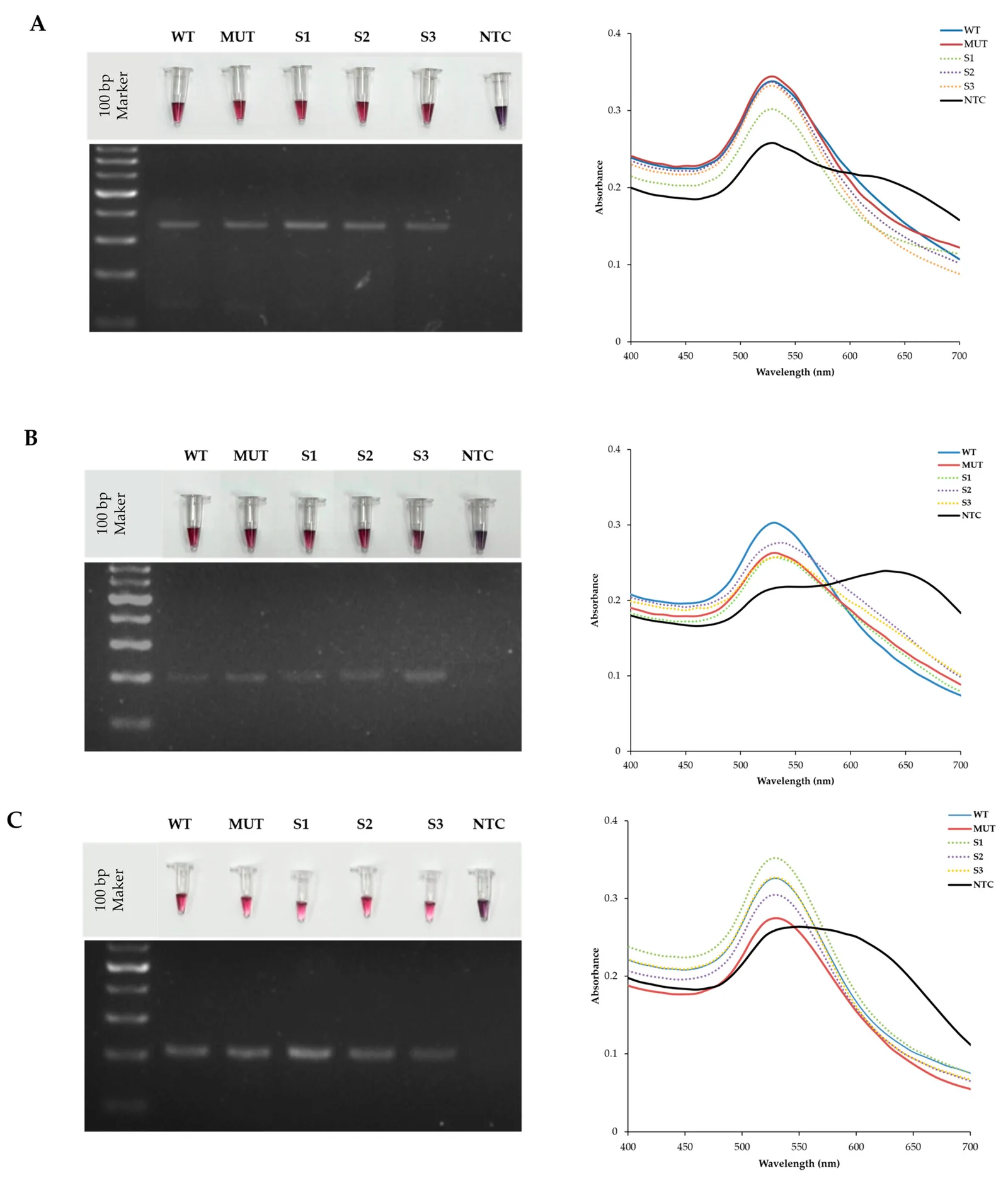
**Internal-control testing of the RR-TB GoldDx and Hr-TB GoldDx assays using DNA from clinical *M. tuberculosis* isolates.** RPA products targeting (**A**) *rpoB*, (**B**) *katG*, and (**C**) the *fabG1–inhA* promoter were detected using unmodified AuNPs. All isolate reactions (S1-S3), as well as the wild-type (WT) and mutant (MUT) controlsremained red following MgCl_2_ addition, confirming successful target amplification, whereas the no-template control (NTC) underwent AuNP aggregation and changed to purple. Agarose gel electrophoresis confirmed amplification products of 359 bp for *rpoB*, 206 bp for *katG*, and 216 bp for the *fabG1–inhA* promoter.

The unmodified-AuNP internal-control tests produced positive results for all 50 isolates, confirming successful amplification of the *rpoB*, *katG*, and *fabG1–inhA* promoter targets (Figure 8). In all valid reactions, the AuNP suspensions remained red after MgCl_2_ addition. Agarose gel electrophoresis independently confirmed amplification products of the expected sizes: 359 bp for *rpoB*, 206 bp for *katG*, and 216 bp for the *fabG1–inhA* promoter. These findings confirmed the presence of the required RPA products before interpretation of the corresponding drug-resistance tests.

#### 3.6.2. Performance of RR-TB GoldDx

**Figure 9:**
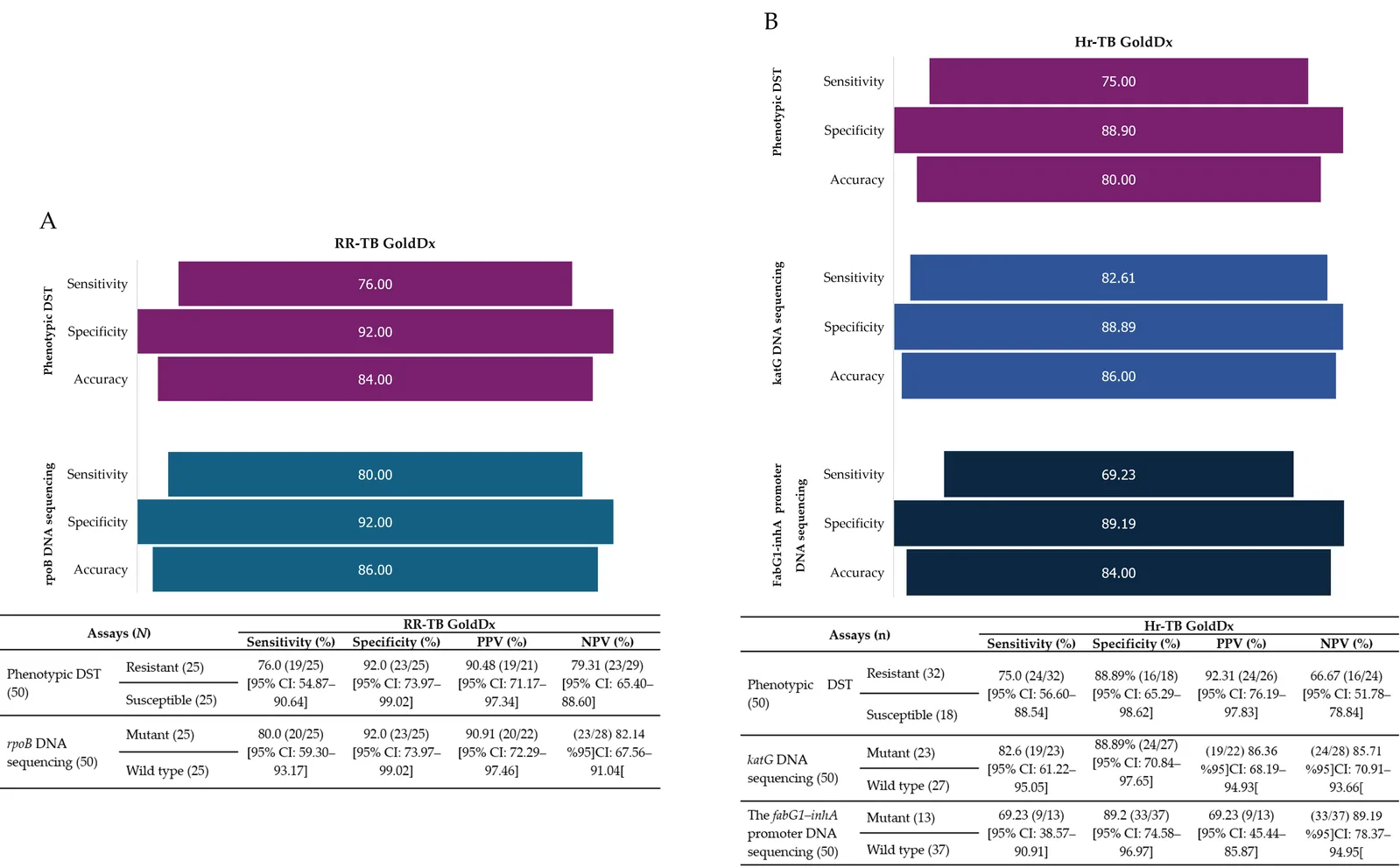
**Diagnostic performance of the GoldDx assays evaluated using 50 clinical *Mycobacterium tuberculosis* isolates.** (**A**) RR-TB GoldDx compared with phenotypic drug susceptibility testing (DST) and *rpoB* DNA sequencing. (**B**) Hr-TB GoldDx compared with phenotypic DST, together with gene-specific DNA sequencing for the *katG* gene and *fabG1–inhA* promoter region.

RR-TB GoldDx was evaluated using 25 rifampicin-resistant and 25 rifampicin-susceptible isolates according to phenotypic DST. The assay results agreed with DST for 42 of 50 isolates, yielding a sensitivity of 76.0%, specificity of 92.0%, positive predictive value of 90.48%, negative predictive value of 79.31%, and overall accuracy of 84.0% (Figure 9A). Cohen’s kappa was 0.68, indicating substantial agreement with phenotypic DST.

When compared with *rpoB* sequencing, RR-TB GoldDx agreed with the reference results for 43 of 50 isolates. The assay showed a sensitivity of 80.0%, specificity of 92.0%, positive predictive value of 90.91%, negative predictive value of 82.14%, and accuracy of 86.0% (Figure 9A). Cohen’s kappa was 0.72, indicating substantial agreement with DNA sequencing.

#### 3.6.3. Performance of Hr-TB GoldDx

Hr-TB GoldDx was evaluated using 32 isoniazid-resistant and 18 isoniazid-susceptible isolates according to phenotypic DST. The assay results agreed with DST for 40 of 50 isolates, yielding a sensitivity of 75.0%, specificity of 88.89%, positive predictive value of 92.31%, negative predictive value of 66.67%, and overall accuracy of 80.0% (Figure 9B). Cohen’s kappa was 0.60, indicating moderate agreement with phenotypic DST (Figure 9B).

For detection of mutations at *katG* codon 315, the assay agreed with DNA sequencing for 43 of 50 isolates. The sensitivity, specificity, positive predictive value, and negative predictive value were 82.61%, 88.89%, 86.36%, and 85.71%, respectively. The overall accuracy was 86.0%, with a Cohen’s kappa of 0.72, indicating substantial agreement with sequencing.

For detection of mutations at position −15 of the *fabG1–inhA* promoter, the assay agreed with sequencing for 42 of 50 isolates. The sensitivity, specificity, positive predictive value, and negative predictive value were 69.23%, 89.19%, 69.23%, and 89.19%, respectively (Figure 9B). The assay achieved an overall accuracy of 84.0%, with a Cohen’s kappa of 0.58, indicating moderate agreement with sequencing.

## 4. Discussion

This study developed and preliminarily evaluated two complementary visual assays, RR-TB GoldDx and Hr-TB GoldDx, for genotypic screening of rifampicin- and isoniazid-resistant *M. tuberculosis*. The platform combines RPA with unmodified AuNPs to confirm successful target amplification and thiol-probe-functionalised AuNPs to detect loss of wild-type sequence recognition at selected resistance-associated loci. Using DNA from 50 clinical *M. tuberculosis* isolates characterised by phenotypic DST and DNA sequencing, all samples produced valid internal-control results. RR-TB GoldDx achieved accuracies of 84% and 86% compared with phenotypic DST and *rpoB* sequencing, respectively, whereas Hr-TB GoldDx achieved an accuracy of 80% compared with phenotypic DST. The individual *katG* codon 315 and *fabG1–inhA* promoter −15 assays achieved accuracies of 86% and 84%, respectively, compared with DNA sequencing. These findings demonstrate the feasibility of obtaining visually interpretable resistance-screening results within approximately 1 h without real-time PCR instrumentation or gel electrophoresis. The principal contribution of this study is therefore the integration of amplification confirmation and wild-type probe-based screening of major rifampicin- and isoniazid-resistance-associated loci within an instrument-minimal RPA–AuNP platform.

The two-component design of the GoldDx platform was intended to improve the reliability of visual interpretation by separating confirmation of target amplification from detection of resistance-associated mutations. AuNPs are suitable visual signal transducers because their localised surface plasmon resonance produces an intense optical response, while changes in interparticle distance markedly affect their absorbance and visible colour. Dispersed AuNPs typically appear red and exhibit a characteristic absorbance band near 520–530 nm, whereas salt-induced aggregation promotes plasmonic coupling, shifts the optical response towards longer wavelengths, and produces a purple or blue colour [15,16]. In the internal-control test, thermally denatured RPA products adsorbed onto unmodified AuNPs and stabilised the particles against MgCl_2_-induced aggregation, whereas reactions lacking amplified DNA underwent aggregation (Figure 2A,B). This behaviour is consistent with the preferential adsorption of ssDNA onto unmodified AuNP surfaces and its ability to protect the particles under increased ionic strength [18]. In the drug-resistance tests, the AuNP surfaces were functionalised with thiolated wild-type probes, taking advantage of the strong affinity between sulphur-containing groups and gold [17]. Complementary wild-type RPA products hybridised with the AuNP-bound probes and maintained colloidal stability, whereas mismatches within the probe-binding regions reduced hybridisation and permitted MgCl_2_-induced aggregation. The internal-control reaction was therefore essential because a purple drug-resistance reaction alone could otherwise represent either loss of wild-type sequence recognition or amplification failure. The different optimal probe and MgCl_2_ concentrations observed among the targets may reflect differences in probe length, nucleotide composition, target–probe hybridisation, probe surface density, and colloidal stability; however, these physicochemical factors were not directly measured in this study. The described AuNP behaviour is consistent with established studies of unmodified and oligonucleotide-functionalised AuNPs [12,15,16,17].

Several nucleic acid amplification test (NAAT)-based assays have been developed for the detection of rifampicin- and isoniazid-resistance-associated mutations in *M. tuberculosis*. Real-time PCR combined with high-resolution melting (HRM) analysis provides relatively broad mutation coverage at comparatively low per-test cost [5,6,7]. Xie et al. [5] reported sensitivities of 93.33% and 95.24% for rifampicin and isoniazid resistance, respectively, with 100% specificity and 98.00% overall concordance with phenotypic DST. The high performance for isoniazid resistance may reflect combined targeting of *katG*, the *ahpC* promoter, and the *inhA* promoter. Similarly, the RIF-RD and INH-RD assays developed by Anukool et al. [6] used multiplex real-time PCR with HRM and standardised ΔTm cutoff values, while the subsequently developed RIF-RDp assay incorporated locked nucleic acid probes to improve detection of selected *rpoB* mutations [7]. Although these assays demonstrated high diagnostic performance and relatively low estimated per-test costs (USD 7–10), they require real-time PCR instrumentation, approximately 2.5–4.2 h to complete, and are generally limited to centralised laboratory settings.

Isothermal amplification methods have also been explored for mutation-based drug-resistance detection. For example, Liu et al. [13] developed an MDR-LAMP assay targeting mutations in *rpoB*, *katG*, and *inhA*, with sensitivity and specificity of 85.5% and 93.6%, respectively, against phenotypic resistance. However, LAMP generally requires multiple primers, operates at approximately 60–65 °C, and generates complex, highly structured amplification products that may complicate downstream hybridisation-based analysis. RPA offers not only a lower-temperature alternative, it also provides multiple options for detection including colourimetric, fluorescence, lateral flow strips, and a CRISPR/Cas-based system [11,24]. Recently, Singpanomchai et al. [14] developed an allele-specific RPA assay combined with SYBR Green I for naked-eye colourimetric detection, with reported limits of detection of 5 ng for mutation-targeting primer sets and 0.05 ng for the IS*1081* primer set. Although sensitivity for rifampicin resistance was 85.19% against phenotypic DST, sensitivity for isoniazid resistance was lower at 59.38%, possibly reflecting the restriction of the assay to *katG* codon 315. In northern Thailand, *katG* S315T has been reported as the predominant *katG* mutation, while −15 C > T is the most common *inhA* promoter mutation. Hr-TB GoldDx therefore targets both *katG* codon 315 and the *fabG1–inhA* promoter −15 position and achieved a sensitivity of 75.0% (24/32; 95% CI: 57.9–86.8) against phenotypic DST.

Broader mutation identification has been achieved by combining RPA with targeted sequence-based analysis. Gliddon et al. [25] established a 90-min RPA protocol to generate relatively long amplicons of approximately 1300–1600 bp, followed by MinION nanopore sequencing for real-time identification of resistance-associated mutations in the *rpoB*, *katG*, and *inhA* regions. Although this approach provides broader mutation-level information, it requires sequencing instrumentation and computer-based analysis and therefore does not provide a direct naked-eye readout or a simple point-of-care format. In addition, TB-GoldDx combined RPA with unmodified AuNPs for rapid visual detection of the *M. tuberculosis* complex using the multicopy IS*6110* target and demonstrated applicability to DNA extracted from sputum specimens [21]. RR-TB GoldDx and Hr-TB GoldDx extend this approach from organism detection to drug-resistance screening by incorporating thiol-functionalised wild-type probes targeting five positions across *rpoB*, *katG*, and the *fabG1–inhA* promoter, while retaining unmodified-AuNP reactions to confirm amplification. Unlike assays designed primarily to establish the presence of a pathogen, the present platform must discriminate single-nucleotide mismatches within single-copy resistance loci. In particular, the multicopy IS*6110* target used in TB-GoldDx may favour greater analytical sensitivity than the single-copy resistance loci examined here [21]. The main contribution of RR-TB GoldDx and Hr-TB GoldDx is therefore not necessarily superior analytical sensitivity, but the combination of visual amplification confirmation with screening of multiple clinically relevant resistance-associated regions in a relatively simple AuNP-based platform. Direct comparisons of diagnostic performance are therefore limited by differences in target copy number, amplification chemistry, detection format, specimen type, comparator method, and study design [21,26,27].

RR-TB GoldDx showed slightly greater agreement with DNA sequencing than with phenotypic DST, achieving accuracies of 86% and 84% and Cohen’s kappa values of 0.72 and 0.68, respectively. This finding is biologically plausible because both RR-TB GoldDx and sequencing examine genetic variation within *rpoB*, whereas phenotypic DST measures bacterial growth at a defined rifampicin concentration. The assay showed higher specificity than sensitivity against both comparator methods, suggesting that isolates retaining the targeted wild-type sequences were generally classified correctly, whereas some resistant or mutant isolates were not detected. The probes target *rpoB* codons 435, 445, and 450 according to the current *M. tuberculosis* numbering system, historically referred to as codons 516, 526, and 531, respectively [6]. These positions are among the most frequently affected codons in rifampicin-resistant *M. tuberculosis*, both globally and in Thailand [6,8,28]. The regional relevance of the targets is supported by studies reporting mutations at these major *rpoB* hotspots among rifampicin-resistant and multidrug-resistant isolates from Thailand [6,28]. However, the wild-type probe format detects reduced complementarity within a probe-binding region rather than identifying a particular nucleotide or amino acid substitution. Its theoretical ability to detect multiple variants at the selected codons should therefore not be interpreted as experimental validation of every reported mutation. The observed performance reflects the mutation spectrum represented among the isolates included in this preliminary study and requires confirmation using larger panels containing diverse, well-characterised *rpoB* variants.

Hr-TB GoldDx achieved an overall accuracy of 80% and moderate agreement with phenotypic DST, with a Cohen’s kappa value of 0.60, sensitivity of 75.0%, and specificity of 88.89%. When evaluated against DNA sequencing, the *katG* codon 315 assay showed slightly higher agreement than the *fabG1–inhA* promoter −15 assay, with accuracies of 86% and 84%, respectively. The lower sensitivity than specificity is consistent with the greater genetic heterogeneity of isoniazid resistance. Although *katG* codon 315 and position −15 of the *fabG1–inhA* promoter are among the most frequently reported isoniazid-resistance determinants, resistance may also result from mutations at other positions within *katG*, elsewhere in the *fabG1–inhA* regulatory region, or in additional genes involved in isoniazid activation, target regulation, and cellular responses to oxidative stress [8,10]. The relevance of the selected targets to northern Thailand is supported by Anukool et al., who reported *katG* S315T as the predominant *katG* mutation and −15 C > T as the most frequent *inhA* promoter mutation among the examined MDR-TB isolates [6]. Isolates carrying resistance-associated mutations outside these two probe-binding regions would nevertheless retain the targeted wild-type sequences and could be classified as susceptible by Hr-TB GoldDx. Consequently, the performance reported here does not indicate complete coverage of the molecular mechanisms underlying isoniazid resistance.

Discordant results between the GoldDx assays and their comparator methods may have arisen from several analytical and biological factors. Because the drug-resistance components use wild-type probes, a purple or blue colour indicates reduced recognition of the wild-type sequence within the probe-binding region rather than direct identification of a specific resistance-associated mutation. Mutations outside the targeted regions would therefore remain undetected, whereas uncommon substitutions or non-resistance-associated polymorphisms within a probe-binding region could reduce hybridisation and produce an apparent mutant result. Heteroresistance or mixed bacterial populations may also affect interpretation because residual wild-type DNA could hybridise with the probe and stabilise the AuNPs despite the presence of a minority mutant population. Differences in template concentration, RPA yield, probe–target hybridisation efficiency, nanoparticle stability, and visual interpretation of borderline red-to-purple reactions could further contribute to variability. Discordance with phenotypic DST may also reflect mutation-specific resistance levels, borderline minimum inhibitory concentrations, or differences in critical concentrations and testing conditions [4]. Conversely, conventional sequencing may fail to detect low-frequency mutant subpopulations. RR-TB GoldDx and Hr-TB GoldDx should therefore be interpreted as screening assays for loss of wild-type recognition within selected resistance-associated regions rather than definitive methods for identifying individual mutations or excluding all mechanisms of rifampicin and isoniazid resistance.

The operational characteristics of RR-TB GoldDx and Hr-TB GoldDx may support their further development for laboratories with limited access to specialised molecular testing. The assays produced visually interpretable results within approximately 1 h, with an estimated reagent cost of THB 500–550 (approximately USD 15–16) per test, and did not require real-time PCR instrumentation, an automated cartridge analyser, or post-amplification gel electrophoresis. In comparison, Xpert MTB/RIF and Xpert MTB/RIF Ultra provide automated sample-to-answer detection of *M. tuberculosis* and rifampicin resistance but require the dedicated GeneXpert platform and do not directly determine isoniazid resistance [4]. First-line line-probe assays can detect resistance-associated mutations in *rpoB*, *katG*, and the *fabG1–inhA* promoter, but their workflow requires DNA extraction, PCR amplification, strip hybridisation, washing, and trained interpretation [4]. The Anyplex II MTB/MDR platform detects *M. tuberculosis* and mutations associated with rifampicin and isoniazid resistance using multiplex real-time PCR and provides objective instrument-based analysis and batch-testing capacity, but it requires real-time PCR equipment and proprietary reagents [29].

The GoldDx platform may consequently provide a lower-instrument alternative for preliminary resistance screening. Relative to previously reported RPA–AuNP systems, it also provides internal amplification confirmation and covers several clinically relevant resistance loci. Nevertheless, the current workflow requires multiple reactions, post-amplification denaturation and liquid-transfer steps, and prepared AuNP reagents, which may reduce ease of use and increase contamination risk compared with closed-cartridge or fully integrated systems. Moreover, the estimated reagent cost excludes DNA extraction, labour, equipment depreciation, quality control, repeat testing, and waste disposal. Because the assays were evaluated using DNA extracted from cultured isolates rather than directly from respiratory specimens, their apparent cost and operational advantages remain preliminary.

Several limitations should be considered when interpreting these findings. First, the assays were evaluated using DNA extracted from only 50 clinical *M. tuberculosis* isolates rather than directly from sputum or other respiratory specimens. Their performance may differ with direct specimens because of lower bacillary loads, variable DNA quality, amplification inhibitors, and mixed microbial or mycobacterial populations. Second, the isolate collection may not represent the full diversity of resistance-associated mutations and *M. tuberculosis* lineages circulating across different geographical regions. Third, mutations outside *rpoB* codons 435, 445, and 450, *katG* codon 315, and position −15 of the *fabG1–inhA* promoter would not be detected. The study also did not determine variant-specific analytical limits of detection, the minimum detectable mutant proportion in wild-type–mutant mixtures, inter-operator reproducibility, batch-to-batch AuNP consistency, reagent shelf life, or objective colour thresholds for borderline reactions. Visual interpretation and post-amplification handling may introduce subjectivity and amplicon-contamination risks. Future studies should therefore include larger multicentre isolate collections representing diverse mutation profiles and phylogenetic lineages, direct evaluation with respiratory specimens, and analytical panels containing well-characterised or synthetic wild-type and mutant targets. Experiments using different mutant-to-wild-type ratios, multiple operators and reagent lots, and smartphone- or spectrophotometer-assisted colour analysis would improve assay standardisation. Lyophilised reagents, closed-tube processing, and integration of amplification and detection into a single disposable device could further improve usability and biosafety. Finally, prospective head-to-head evaluations against WHO-recommended rapid molecular tests, line-probe assays, Anyplex, DNA sequencing, and phenotypic DST, together with formal workflow and cost-effectiveness analyses, are required before RR-TB GoldDx and Hr-TB GoldDx can be considered for routine clinical implementation.

## 5. Conclusions

RR-TB GoldDx and Hr-TB GoldDx were successfully developed as rapid visual RPA–AuNP assays for screening major rifampicin- and isoniazid-resistance-associated mutations in *M. tuberculosis*. The platform combines an unmodified-AuNP internal-control reaction for confirmation of target amplification with thiol-probe-functionalised AuNP reactions for detection of loss of wild-type sequence recognition at selected loci in *rpoB*, *katG*, and the *fabG1–inhA* promoter. A principal novelty of this platform is the integration of visual amplification confirmation with wild-type loss detection within a low-temperature RPA–AuNP workflow. Importantly, this strategy does not require a mutation-specific probe for every individual variant; instead, loss of wild-type probe recognition can potentially indicate multiple resistance-associated substitutions occurring within the interrogated probe regions, thereby providing broader mutation coverage for RR-TB and Hr-TB screening while retaining a simple visual readout. Using DNA from 50 clinical *M. tuberculosis* isolates characterised by phenotypic DST and DNA sequencing, the assays produced interpretable results within approximately 1 h and showed moderate-to-substantial agreement with the comparator methods. RR-TB GoldDx demonstrated 80.00% sensitivity and 92.00% specificity against DNA sequencing, while the *katG* and *fabG1–inhA* components of Hr-TB GoldDx showed sensitivities of 82.61% and 69.23% and specificities of 88.89% and 89.19%, respectively. The assays require limited instrumentation and have an estimated reagent cost of THB 500–550 per test. These findings support their potential as preliminary drug-resistance screening tools for laboratories with limited access to advanced molecular platforms. However, the assays currently detect only selected resistance-associated regions and were evaluated using DNA extracted from cultured isolates. Larger multicentre studies using genetically diverse isolates and direct respiratory specimens, together with analytical, reproducibility, workflow, and cost-effectiveness evaluations, are required before clinical implementation.

## Figures and Tables

**Table 1:** Selected RPA primers and wild-type thiol probes used in RR-TB GoldDx and Hr-TB GoldDx.

Assay	Target	Primers/Probe Name	Genomic Position	Amplicon/Probe Length	Resistance-Associated Locus ^1^
RR-TB GoldDx	*rpoB*	RPA-*rpoB*-F9	760,930–760,956	27	Codon 516, 526, 531
		RPA-*rpoB*-R4	761,263–761,288	26	Codon 516, 526, 531
		Thiol-rpoB 516 wt	761,098–761,124	26	Codon 516
		Thiol-*rpoB* 526 wt	761,125–761,149	25	Codon 526
		Thiol-*rpoB* 531 wt	761,147–761,167	21	Codon 531
Hr-TB GoldDx	*katG*	RPA-*katG*-F9	2,155,026–2,155,052	27	Codon 315
		RPA-*katG*-R9	2,155,202–2,155,229	28	Codon 315
		*katG* 315 wt	2,155,152–2,155,177	26	Codon 315
	the *fabG1–inhA* promoter	RPA-*inhA*-F11	1,673,285–1,673,310	26	Position −15
		RPA-*inhA*-R8	1,673,474–1,673,500	27	Position −15
		Thiol-inhA −15 wt	1,673,416–1,673,435	20	Position −15

^1^ Codons 516, 526, and 531 correspond to codons 435, 445, and 450, respectively, according to the current *M. tuberculosis* numbering system.

## Data Availability

The original contributions presented in this study are included in the article. Further inquiries can be directed to the corresponding author.

## Abbreviations

The following abbreviations are used in this manuscript:

AuNPs	Gold nanoparticles
DR-TB	Drug-resistant tuberculosis
DST	Drug-susceptibility testing
Hr-TB	Isoniazid-resistant tuberculosis
Hr-TB GoldDx	Gold nanoparticle-based assay for detecting isoniazid-resistance-associated mutations
INH	Isoniazid
LPA	Line-probe assay
MDR-TB	Multidrug-resistant tuberculosis
NTC	No-template control
RPA	Recombinase polymerase amplification
RR-TB	Rifampicin-resistant tuberculosis
RR-TB GoldDx	Gold nanoparticle-based assay for detecting rifampicin-resistance-associated mutations
RRDR	Rifampicin resistance-determining region
UV–Vis	Ultraviolet–visible spectrophotometry

## Appendix A

**Table A1:** Reported *rpoB* amino-acid substitutions associated with rifampicin resistance that fall within the binding regions of the three RR-TB GoldDx wild-type probes targeting codons 435, 445, and 450 of *Mycobacterium tuberculosis*.

RR-TB GoldDx Probe	Current *M. tuberculosis* Codon	Historical Codon	Wild-Type Residue	Reported Amino-Acid Substitution	WHO Confidence Grading
Thiol-*rpoB* 516 wt	435	516	Asp	Asp435Ala	Associated with resistance—interim
Thiol-*rpoB* 516 wt	435	516	Asp	Asp435Asn	Associated with resistance—interim
Thiol-*rpoB* 516 wt	435	516	Asp	Asp435Glu	Associated with resistance—interim
Thiol-*rpoB* 516 wt	435	516	Asp	Asp435Gly	Associated with resistance—interim
Thiol-*rpoB* 516 wt	435	516	Asp	Asp435His	Associated with resistance—interim
Thiol-*rpoB* 516 wt	435	516	Asp	Asp435Leu	Associated with resistance—interim
Thiol-*rpoB* 516 wt	435	516	Asp	Asp435Phe	Associated with resistance
Thiol-*rpoB* 516 wt	435	516	Asp	Asp435Tyr	Associated with resistance
Thiol-*rpoB* 516 wt	435	516	Asp	Asp435Val	Associated with resistance
Thiol-*rpoB* 526 wt	445	526	His	His445Arg	Associated with resistance
Thiol-*rpoB* 526 wt	445	526	His	His445Asn	Associated with resistance
Thiol-*rpoB* 526 wt	445	526	His	His445Asp	Associated with resistance
Thiol-*rpoB* 526 wt	445	526	His	His445Cys	Associated with resistance
Thiol-*rpoB* 526 wt	445	526	His	His445Gln	Associated with resistance—interim
Thiol-*rpoB* 526 wt	445	526	His	His445Gly	Associated with resistance—interim
Thiol-*rpoB* 526 wt	445	526	His	His445Leu	Associated with resistance
Thiol-*rpoB* 526 wt	445	526	His	His445Phe	Associated with resistance—interim
Thiol-*rpoB* 526 wt	445	526	His	His445Pro	Associated with resistance—interim
Thiol-*rpoB* 526 wt	445	526	His	His445Ser	Associated with resistance
Thiol-*rpoB* 526 wt	445	526	His	His445Thr	Associated with resistance—interim
Thiol-*rpoB* 526 wt	445	526	His	His445Tyr	Associated with resistance
Thiol-*rpoB* 531 wt	450	531	Ser	Ser450Ala	Associated with resistance—interim
Thiol-*rpoB* 531 wt	450	531	Ser	Ser450Cys	Associated with resistance—interim
Thiol-*rpoB* 531 wt	450	531	Ser	Ser450Gln	Associated with resistance—interim
Thiol-*rpoB* 531 wt	450	531	Ser	Ser450Gly	Associated with resistance—interim
Thiol-*rpoB* 531 wt	450	531	Ser	Ser450Leu	Associated with resistance
Thiol-*rpoB* 531 wt	450	531	Ser	Ser450Met	Associated with resistance—interim
Thiol-*rpoB* 531 wt	450	531	Ser	Ser450Phe	Associated with resistance
Thiol-*rpoB* 531 wt	450	531	Ser	Ser450Trp	Associated with resistance
Thiol-*rpoB* 531 wt	450	531	Ser	Ser450Tyr	Associated with resistance
Thiol-*rpoB* 531 wt	450	531	Ser	Ser450Val	Associated with resistance—interim

**Note:** The listed substitutions were compiled from the WHO mutation catalogue [5]. Codons 435, 445, and 450 correspond to historical codons 516, 526, and 531, respectively. Theoretical coverage indicates that the substitutions occur within the probe-targeted codons and does not imply experimental validation of every variant.
